# Do multiple experimenters improve the reproducibility of animal studies?

**DOI:** 10.1371/journal.pbio.3001564

**Published:** 2022-05-05

**Authors:** Vanessa Tabea von Kortzfleisch, Oliver Ambrée, Natasha A. Karp, Neele Meyer, Janja Novak, Rupert Palme, Marianna Rosso, Chadi Touma, Hanno Würbel, Sylvia Kaiser, Norbert Sachser, S. Helene Richter

**Affiliations:** 1 Department of Behavioural Biology, University of Münster, Münster, Germany; 2 Otto Creutzfeldt Center for Cognitive and Behavioral Neuroscience, University of Münster, Münster, Germany; 3 Department of Behavioural Biology, University of Osnabrück, Osnabrück, Germany; 4 Data Sciences & Quantitative Biology, Discovery Sciences, R&D, AstraZeneca, Cambridge, United Kingdom; 5 Division of Animal Welfare, University of Bern, Bern, Switzerland; 6 Department of Biomedical Sciences, University of Veterinary Medicine, Vienna, Austria; Leiden University Medical Center: Leids Universitair Medisch Centrum, NETHERLANDS

## Abstract

The credibility of scientific research has been seriously questioned by the widely claimed “reproducibility crisis”. In light of this crisis, there is a growing awareness that the rigorous standardisation of experimental conditions may contribute to poor reproducibility of animal studies. Instead, systematic heterogenisation has been proposed as a tool to enhance reproducibility, but a real-life test across multiple independent laboratories is still pending. The aim of this study was therefore to test whether heterogenisation of experimental conditions by using multiple experimenters improves the reproducibility of research findings compared to standardised conditions with only one experimenter. To this end, we replicated the same animal experiment in 3 independent laboratories, each employing both a heterogenised and a standardised design. Whereas in the standardised design, all animals were tested by a single experimenter; in the heterogenised design, 3 different experimenters were involved in testing the animals. In contrast to our expectation, the inclusion of multiple experimenters in the heterogenised design did not improve the reproducibility of the results across the 3 laboratories. Interestingly, however, a variance component analysis indicated that the variation introduced by the different experimenters was not as high as the variation introduced by the laboratories, probably explaining why this heterogenisation strategy did not bring the anticipated success. Even more interestingly, for the majority of outcome measures, the remaining residual variation was identified as an important source of variance accounting for 41% (CI_95_ [34%, 49%]) to 72% (CI_95_ [58%, 88%]) of the observed total variance. Despite some uncertainty surrounding the estimated numbers, these findings argue for systematically including biological variation rather than eliminating it in animal studies and call for future research on effective improvement strategies.

## Abstract 2

An experimenter heterogenisation was not sufficient to prevent idiosyncratic results in a multi-laboratory setting. Astonishingly, neither the experimenter nor the laboratory accounted for the main portion of the observed variation, but a high amount of residual variation in fact remained unexplained despite strict standardisation regimes.

## Introduction

One core principle of science is the reproducibility of results, i.e., obtaining consistent results when replicating a study by collecting new data. However, over the past decade, the credibility of scientific results has been challenged by the fact that many replication studies failed to reproduce published results [1–4]. Based on such replication studies, the prevalence for irreproducible results was estimated to range between 50% and 90% and the majority of scientists in the life sciences are convinced that this “reproducibility crisis” needs to be addressed [1,5].

Failures to reproduce results of previous studies have been attributed to deficiencies in reporting standards, as well as to flaws in experimental design and statistical analysis [6–10]. To improve the situation, several guidelines such as the TOP [11], ARRIVE [12,13], and PREPARE [14] guidelines have been established to improve the planning, conduct, analysis, and reporting of studies. All of these attempts were already successful in increasing the overall quality of reported studies [15]. However, these strategies assume that the issue of irreproducibility can be comprehensively solved, provided that the experiment is planned and conducted with adequate expertise and that the methods are transparently reported in sufficient detail. With respect to animal research, one additional cause of irreproducibility is thereby often neglected or not adequately addressed. Every study involving animals is challenged by the fact that living organisms are highly responsive to their environment. This flexibility in the phenotype of an animal with a specific genotype towards different environmental cues is known as phenotypic plasticity [16].

Phenotypic plasticity leads to variation in results, even if all animals are genetically identical [17,18]. Plastic responses of an organism with a specific genotype towards its local environment (i.e., the laboratory environment) may result in remarkably different results across replicate studies [19]. This was impressively demonstrated by Crabbe and colleagues [20]. Despite extensive standardisation of the experimental conditions across 3 laboratories, they obtained conflicting results in behavioural differences between 8 mouse strains across the 3 laboratories. The authors concluded that small divergences in the local environment of the laboratories modulated the effects of genotype on behaviour, leading to idiosyncratic results. It was suggested that these divergences were most likely due to differences between the experimenters [21]. Indeed, also other studies produced remarkably different results when the animals were tested by different experimenters [22–24]. Even single aspects of the experimenter identity, such as the sex of the experimenter [22] or the way an animal is handled [25,26], have been identified to significantly affect the outcome. Furthermore, the importance of the factor “experimenter” was convincingly illustrated by a meta-analysis on acute pain sensitivity, showing that the experimenter accounted for more variance than any other known factor [27]. Thus, although it is not yet understood completely, why the factor “experimenter” has such a strong influence, it represents one of the top confounding factors in animal research.

The common approach to dealing with such factors is the rigorous standardisation of the experimental conditions. Thus, in most studies, all animals have the same age, are housed under the same conditions, and are tested by the same experimenter. This way, the variation within a study is expected to be minimised, and thus, the power to detect potential treatment effects is enhanced. However, this concept relies on the assumption of a fixed treatment effect, which can be detected by eliminating all sources of variation within a study. It ignores the fact that biological variation is an inherent characteristic of animal research and treatment effects may vary depending on the exact conditions to which a study is standardised [19,28]. Although diverging findings might provide novel insights about a phenomenon under investigation, meaningful conclusions can only be drawn if the exact differences between experiments are known. Researchers that standardise their study conditions rigorously to a set of often unknown factors (i.e., noise level, personnel), however, limit the inference space of the study to these narrowly defined conditions, thereby hampering the detection of such potentially meaningful study differences. Therefore, rigorous standardisation is at risk to produce idiosyncratic results that represent local “truths” and are often not reproducible.

This fallacy of enhancing reproducibility through standardisation becomes most apparent with respect to the experimenter effect: It is not only impossible to standardise experimenters across laboratories, but fundamentally misleading as the experimenter identity usually is of no biological interest for the study question. Instead of minimising variation by rigorous standardisation, embracing biological variability might be a better strategy to address replication failure. With regard to the experimenter, this would mean that instead of trying to eliminate this uncontrollable influencing factor in science, it could be used in a controlled way to systematically introduce variation in a study [29].

A growing body of evidence suggests that introducing heterogeneity in a controlled way to the study design (referred to as “systematic heterogenisation”) increases the inference space of the results, leading to better reproducibility under varying background conditions [28,30–35]. For example, splitting an experiment into several batches (i.e., mini-experiments with slightly varying conditions) improved the reproducibility of findings across replicate experiments within the same laboratory [36]. Whereas such data provide a convincing proof-of-concept of systematic heterogenisation, empirical evidence from tests across independent laboratories is still limited [33,37,38]. Therefore, further studies are urgently needed that (1) identify potential heterogenisation factors; and (2) empirically validate such strategies in a real-life situation. A successful and versatile heterogenisation strategy thereby comprises 2 different characteristics. First, the strategy needs to introduce sufficient variation in the study design to mimic the variation that usually occurs between studies. Second, the factor used to introduce variation should not be in the focus of the study question itself (i.e., is of no biological interest). In light of the discussion summarised above, the factor “experimenter” complies with both requirements and thus represents a promising factor for a heterogenisation strategy.

Against this background, the overall aim of the present multilaboratory study was to empirically test the potential of the experimenter as a heterogenisation factor. We expected improved reproducibility of research findings in comparison to a conventionally standardised design. In line with this assumption, we also aimed at estimating the amount of variance explained by multiple experimenters, assuming that the factor “experimenter” represents the major source of variation. To this end, the same animal experiment was replicated independently in 3 different laboratories, using both a heterogenised design with 3 experimenters being involved in testing the study sample and a standardised design with one experimenter testing the study sample. To assess reproducibility, a typical animal experiment in the field of biomedical research was mimicked. More precisely, many studies examine the role of specific genes in the modulation of the phenotype and therefore rely on the phenotypic characterisation of animals of different genotypes. To reflect such an experiment with a typical “treatment under investigation”, 2 inbred mouse strains (i.e., different genotypes) were tested in a range of physiological and behavioural outcome variables commonly used in such phenotyping studies (cf. [20]).

## Methods

### Animals and housing conditions

In this study, 96 naïve female mice of 2 inbred mouse strains (C57BL/6J and DBA/2N, 48 mice per strain) were used in each of the 3 laboratories. To ensure consistent housing conditions across the 3 laboratories, female mice were chosen because, in contrast to male mice, they can be easily housed in stable groups without taking the risk of having to separate some mice over the course of the experimental phase (for a discussion about housing male mice, see [39,40]). All animals were provided by the same commercial supplier (Charles River Laboratories). As each experimenter conducted the experiment in an independent batch, animals were delivered for all experiments separately at an age of 7 weeks (for details, see S2 Table). Upon arrival, the animals were housed in same strain groups of 2 mice per cage. All animals of one experiment (12 per strain) were housed in the same rack, beginning at the top with the strains being allocated to their horizontal and vertical rack position in a balanced way. The allocation to the rack position was the same for all experiments and was harmonised across laboratories. The animals were housed according to laboratory-specific housing protocols (for details on, e.g., cage type, bedding material, and temperature, see S1 Table). Food pellets and tap water were provided ad libitum. Cages were cleaned weekly and housing rooms were maintained at a reversed 12/12 h light–dark cycle in all 3 laboratories.

### Ethics statement

All procedures complied with the regulations covering animal experimentation within Germany (Animal Welfare Act), Switzerland (Swiss Animal Welfare Ordinance TSchV 455.1) and the EU (European Communities Council DIRECTIVE 2010/63/EU) and were approved by the local (Gesundheits- und Veterinäramt Münster, Nordrhein-Westfalen) and federal authorities (Landesamt für Natur, Umwelt und Verbraucherschutz Nordrhein-Westfalen “LANUV NRW”, reference number 84–02.04.2015.A245, Niedersächsisches Landesamt für Verbraucherschutz und Lebensmittelsicherheit “LAVES Niedersachsen”, reference number 33.19-42502-04-19/3222 and Cantonal Veterinary Office in Bern, Switzerland, permit number: BE 81/18).

### Concept of the study

The present project was designed as a multilaboratory study, involving 3 independent laboratories in Germany and Switzerland (A: Veterinary Public Health Institute, Animal Welfare Division, University of Bern, Switzerland; B: Department of Behavioural Biology, University of Münster, Germany and C: Department of Behavioural Biology, University of Osnabrück, Germany). The overall aim was to compare a conventionally standardised design and a systematically heterogenised design in a real-life situation (cf. [33]). Therefore, each of 3 laboratories tested 2 inbred mouse strains (C57BL/6J and DBA/2N) for a variety of different behavioural and physiological outcome measures.

**Fig 1:**
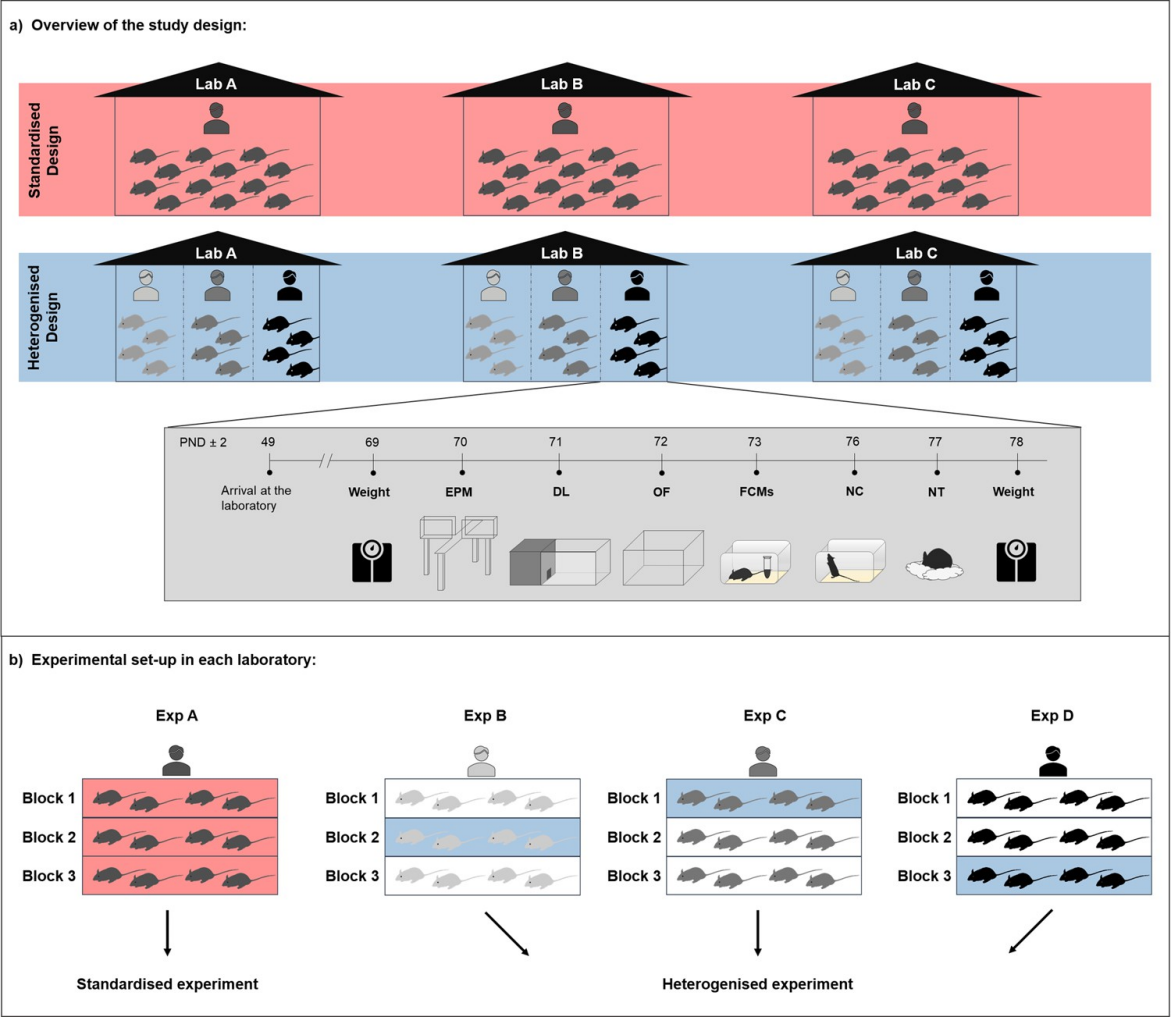
**(a) Overview of the study design.** To examine reproducibility of research findings across laboratories, the same animal experiment was independently repeated in 3 laboratories in both a conventionally standardised (red) and a heterogenised design (blue). In the standardised design, mice of 2 strains, C57BL/6J and DBA/2N (*n =* 12 per strain), were tested by one experimenter, while in the heterogenised design, 3 different experimenters were involved in conducting the experiment (i.e., 4 mice per strain and experimenter were included in the analysis). Each experimenter, regardless of the design, conducted the same animal experiment following the same standardised protocols. All test procedures were conducted in the same order for all animals. (**b) Experimental setup in each laboratory.** In each laboratory, 4 experimenters conducted the same animal experiment as described above. All 4 experiments were conducted according to a randomised block design including 3 blocks per experiment (Block 1–3). In each block, mice were housed in the same row in the same rack (e.g., top, middle, bottom) and, thus, these animals shared the same environmental background with respect to lighting conditions, humidity, and temperature. Out of these 4 experiments per laboratory, one was randomly selected and classified as standardised experiment (e.g., Exp A, red). For the heterogenised design, one block (i.e., 4 mice per strain) from each of the remaining 3 experimenters (Exp B–Exp D) was pseudo-randomly selected and classified as part of a heterogenised experiment. This was done in a way that in each heterogenised experiment all 3 blocks were represented. Please note: Shown is one example of a possible allocation of experimenters and blocks to the standardised and heterogenised design, respectively (for details, see “Data analysis” and S3 Table). Furthermore, only mice of one strain (half of the mice tested) are visualised in the figure. DL, Dark Light; EPM, Elevated Plus Maze; FCMs, faecal corticosterone metabolites; NC, Novel Cage; NT, Nest; OF, Open Field; PND, postnatal day.

In contrast to the standardised design, the heterogenised design included variation among different experimenters in each laboratory. In detail, in the standardised design, all mice of one experiment (*n =* 12 mice per strain, see “Data analysis” for details on sample size estimation) were tested by one experimenter, while in the heterogenised design, 3 different experimenters were involved in conducting the experiment (i.e., 4 mice per strain and experimenter were included in the analysis; see Fig 1A). This allocation of the experimenters to the 2 designs was randomly chosen before the study was conducted using the randomisation software “Research Randomizer” [41].

To evaluate the reproducibility of the results in both designs and the factors that contribute the most to the variance in the data, 3 different analyses were applied: (1) reproducibility of the strain effect across laboratories; (2) exploration of impact of experimenter allocation; and (3) components of variance analysis.

In the first analysis, we compared the reproducibility of the results between both designs in terms of the consistency across the 3 laboratories and the performance of the experimental designs to predict the overall effect size. This analysis was completed on the basis of the prior determined experimenter allocation. In the second analysis, we evaluated if the results of the first analysis may have been due to the specific experimenters allocated to each design rather than due to the characteristics of the designs itself. Here, the first analysis was repeated on the basis of 10 alternative experimenter allocations (see “Experimental setup in each laboratory” and “Data analysis” for details).

In a third analysis, we used the data generated to disentangle the different components of variance and estimate their contribution to the total variance in this study (see “Data analysis” for details).

### Experimental setup in each laboratory

In each of the 3 participating laboratories, 4 experimenters conducted the same animal experiment (C57BL/6J versus DBA/2N mice) following the same standardised test protocols (see “Experimental procedures”). In the laboratory in Bern, all experimenters were females, whereas in Münster and Osnabrück, male and female experimenters were involved in the study. Experimenters with different levels of experience in both, handling mice and conducting the test procedures, were involved in all laboratories (for details, see S2 Table). This diversity was deliberately chosen to cover a wide range of potential influencing factors, which are considered to account for experimenter effects (e.g., sex and experience [22,23]).

Theoretically, one experimenter per laboratory had to test a “full” experiment of 12 mice per strain in the standardised design, whereas the 3 experimenters allocated to the heterogenised design only needed to test a reduced number of 4 mice per strain. However, in practice, all experimenters tested the same number of animals as needed in one conventional standardised experiment (i.e., 12 mice per strain) to guarantee that all experimenters were blind to the experimental design. As a consequence, 4 “full” independent experiments were performed by 4 experimenters consecutively in each laboratory (for an overview of the results of all 4 experimenters in each laboratory, see S5–S14 Figs and S8 Table). Each experimenter tested 12 mice per strain (*n =* 12), except for the laboratory in Osnabrück. Here, due to technical reasons, the sample size of 2 experimenters (Exp A and B) was reduced to *n* = 11 for the “C57BL/6J” group and *n* = 9 and *n* = 8, respectively, for the “DBA/2N” group. Due to the consecutive testing of the 4 experimenters, experiments in the heterogenised design comprised not only animals tested by different experimenters, but also tested at different time points, while in the standardised design, all animals from one laboratory were delivered and tested at one specific point in time by one experimenter (Fig 1). Besides of providing blindness, the approach of testing 4 “full” experiments in each laboratory offered the opportunity to simulate different combinations regarding the allocation of the experimenters to the experimental designs (see “Data analysis” and S3 Table).

All “full” experiments were organised according to a randomised block design, where each experiment was divided into 3 “blocks” containing 4 mice per strain (see Fig 1B). Mice of one block were housed in the same row in the same rack (e.g., top, middle, bottom) and thus shared the same “microenvironment” with respect to lighting conditions, humidity, and temperature. This randomised block design provided the basis for the selection process of the animals to the experimental designs. More specifically, one “full” experiment (i.e., 3 blocks, tested by one experimenter) was randomly selected and assigned to the standardised design. For the heterogenised design, one block (i.e., 4 mice per strain) from each of the 3 remaining experimenters was pseudo-randomly selected and classified as part of one heterogenised experiment. Consequently, each selected heterogenised experiment contained 3 blocks tested by 3 different experimenters.

### Experimental procedures

To examine reproducibility of behavioural and physiological differences between the 2 inbred mouse strains (C57BL/6J and DBA/2N), all mice were subjected to the same testing procedures. Thereby, behavioural paradigms were chosen in accordance with established protocols for the phenotypic characterisation of mice in experimental animal research [42].

Experimental procedures started on postnatal day (PND) 70 ± 2 and lasted for 8 days. Over the course of this period, the following tests were conducted during the active phase in the same order for all animals: Elevated Plus Maze (EPM) on PND 70 ± 2, Dark Light (DL) test on PND 71 ± 2, Open Field (OF) test on PND 72 ± 2, Novel Cage (NC) test on PND 76 ± 2, and Nest (NT) test starting on PND 77 ± 2 (see Fig 1). Additionally, faecal samples to determine levels of glucocorticoid metabolites non-invasively were collected on PND 73 ± 2 and the change in body weight over the course of the test phase, from PND 69 ± 2 to PND 78 ± 2, was measured. Details on the experimental procedures are given in S1 Text. Due to technical reasons, the body weight data of 3 mice are missing.

Four days before the start of the experimental phase, habituation of the animals to the designated experimenter took place. Each experimenter handled the mice at least 3 times before the start of the first test procedure (EPM). Animals tested by one experimenter were exclusively handled by this person during the whole experimental phase.

The order of mice tested on the same day was pseudo-randomised, following 2 rules. First, mice of neighbouring cages (C57BL/6J and DBA/2) were tested consecutively. Second, in the EPM, DL, OF, and NC tests, a break of at least 1 h between the testing of 2 cage mates was ensured to minimise influences caused by any disturbances in the cage.

While the experimenters were blind with respect to the allocation of the mice to the experimental design (conventionally standardised or heterogenised design), blinding to the mouse strain was not possible due to different fur colours of the 2 strains (C57BL/6J mice: black, DBA/2N mice: brown). However, the main outcome (i.e., reproducibility of strain differences across laboratories) was unlikely to be influenced by the lack of blinding at this stage of the study. Importantly, all experimenters were unaware of the results from other experimenters in their own laboratory and in the other participating laboratories.

### Data analysis

In this study, 20 outcome measures were recorded, which derived from 7 experimental test procedures. To minimise dependencies between these outcome measures, all 20 outcome measures were checked for correlations among each other. We selected 10 outcome measures (see S4 Table and S4 Fig), which had a correlation coefficient <0.5 and were therefore not highly correlated to each other (cf. [36,37]). To avoid any biases in the selection process, the whole selection process was completed by an experimenter blind to the specific outcome measures. Outcome measures for exclusion were determined in a way that as few outcome measures as possible had to be excluded in this process. Whenever only 2 outcome measures were correlated with each other, it was randomly chosen which one was excluded. The whole selection process was conducted on the basis of the full data set, irrespective of the allocation of the experimenters to the experimental designs and before the following analyses (see below) were carried out.

To estimate the sample size in our study, an a priori power analysis was conducted using previously published data by Bodden and colleagues [31]. According to this data, we expected large effect sizes for the primary outcome of interest: the strain-by-laboratory interaction. With a sample size of *n* = 12 mice per strain and laboratory, we could ensure to detect biologically relevant variations of the strain effect across the 3 laboratories with a power of 80%.

#### Reproducibility of the strain effect across laboratories

The main analysis to evaluate the reproducibility of the treatment effect across the 3 laboratories and compare it between both experimental designs was adapted from von Kortzfleisch and colleagues [36] and comprised the following 2 approaches: (I.) calculating the consistency of the strain effect across laboratories; and (II.) estimating how often and how accurately the overall effect was predicted in the laboratories.

**(I.) Consistency of the strain effect across laboratories**. The consistency of the strain effect across laboratories is statistically reflected in the variation captured by the interaction between the factors “strain” and “laboratory” (i.e., “strain-by-laboratory” interaction). To assess this “strain-by-laboratory” interaction term as a measurement for reproducibility, a linear mixed model (LMM) was applied to both designs (standardised and heterogenised). Details on this analysis and the model equation are presented in S2 Text and S5 Table.

**(II.) Estimation of how often and how accurately the overall effect is predicted in the laboratories**. How good each experimental design predicted the overall effect size was assessed by the following 2 measurements that were adapted from the analysis by Voelkl and colleagues [32]: The coverage probability (Pc) and the proportion of accurate results (Pa). The Pc was assessed by counting how often the effect size estimates from the 3 laboratories and their corresponding confidence intervals (CI_95_) cover the overall effect size. The Pa was determined by counting how often the standardised and heterogenised experiments in the different laboratories predicted the overall effect size accurately with respect to its statistical significance. This was done for each experimental design separately. For details on this analysis, see S2 Text.

#### Impact of experimenter allocation

Regarding the allocation of the experimenters to the 2 experimental designs in each laboratory, several combinations were possible. It might be plausible that the results are dependent on the specific experimenters selected for each experimental design. Therefore, to gain more confidence in the conclusion when comparing both experimental designs, we repeated the previously outlined analyses (I. and II.) for 10 randomly chosen, alternative allocations of the experimenters to the experimental designs (for details, see S3 Table).

#### Components of variance

To assess the influence of the different laboratories and experimenters, we conducted a follow-up analysis, where the data of all mice tested by each experimenter in all 3 laboratories were combined and the proportion of variation due to different sources was calculated. In detail, the total amount of variation in this dataset can be separated into the between-strain variability, between-laboratory variability, between-experimenter variability, strain-by-laboratory interaction variability, strain-by-experimenter interaction variability, between-block variability, strain-by-block interaction variability, between-cage variability, and between-individual variability (residuals). Therefore, the proportion of variance attributable to each factor was estimated using an LMM (see S2 Text).

Nearly all statistical analyses were conducted and graphs created using the statistical software “R” [43] (Version 4.0.2). Only testing for the correlation among outcome measures was done using the statistical software “IBM SPSS Statistics” (IBM Version 23), and the power calculation was done using the statistical software G*Power [44]. Differences were considered to be statistically significant when *p* ≤ 0.05.

## Results

### Reproducibility of the strain effect across laboratories

**Fig 2:**
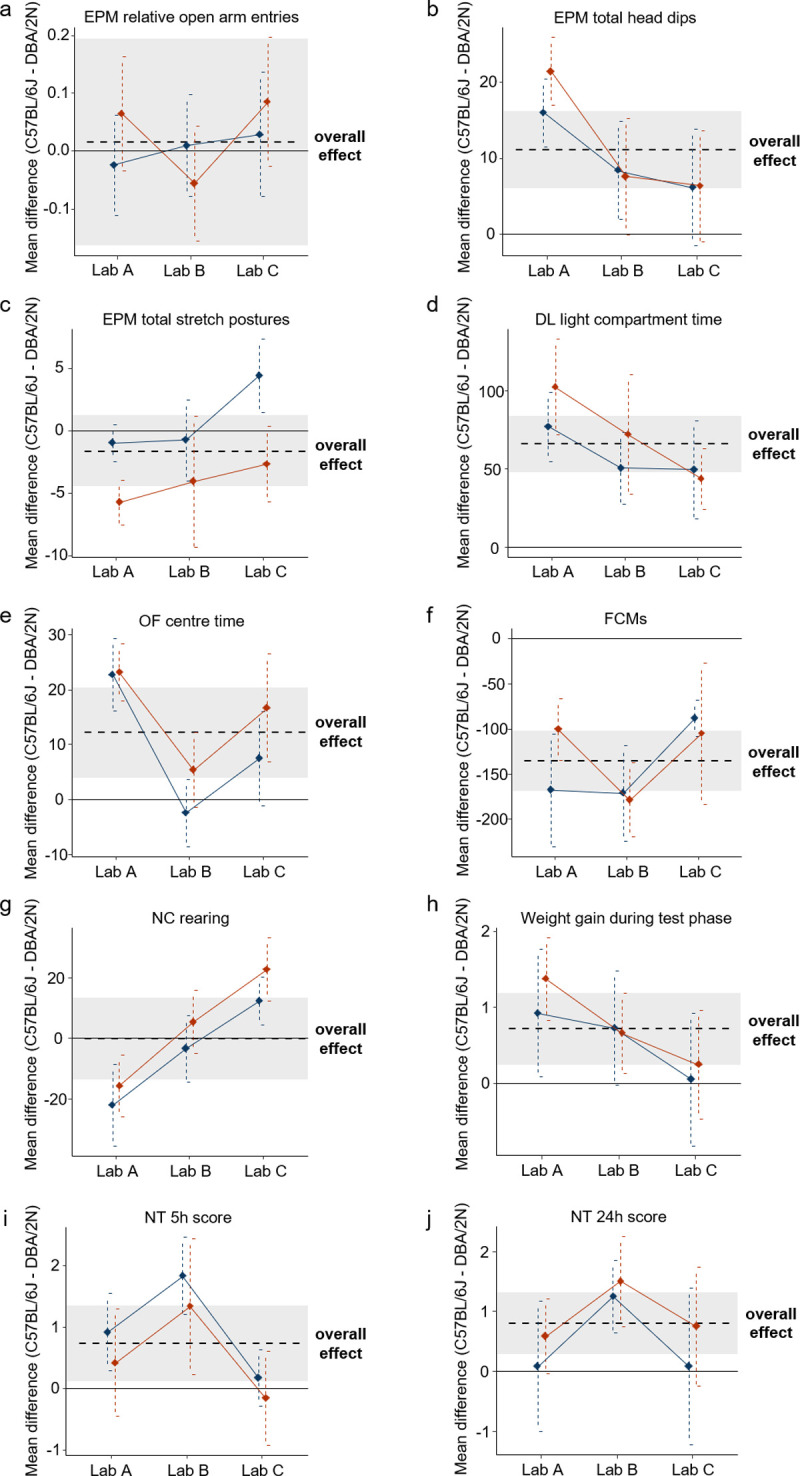
Variation of mean strain differences across the 3 laboratories in the standardised (red) and the heterogenised design (blue). Shown are the mean strain differences of C57BL/6J and DBA/2N mice in the order of testing in (a) “relative open arm entries in the EPM” (b) “total head dips in the EPM” (c) “total stretched postures in the EPM” (d) “light compartment time in the DL test” (e) “OF centre time” (f) “FCMs concentrations” (g) “amount of rearings in the NC test” (h) “weight gain during test phase” (i) “NT test score after 5 h” and (j) “NT test score after 24 h”. The black dashed line and the shaded area indicate the overall mean strain difference of this outcome measure and its corresponding CI_95_. The black solid line reflects a null effect. Dots and vertical dashed lines reflect the mean strain differences and corresponding CI_95_ of the results from the 3 laboratories in each design. The raw data underlying this figure are available in the Figshare repository https://figshare.com/s/f327175aa8b541ef01bd. CI_95_, 95% confidence interval; DL, Dark Light; EPM, Elevated Plus Maze; FCMs, faecal corticosterone metabolites; NC, Novel Cage; NT, Nest; OF, Open Field.

Descriptively, some of the detected strain effects in this study could be well reproduced at all 3 sites, but for others, we found remarkably different results between the 3 laboratories (Fig 2). For example, all 3 laboratories detected a significant strain effect (i.e., CI_95_ intervals distinct from 0) regarding the time spent in the light compartment in the DL with C57BL/6J mice spending more time in the light compartment than DBA/2N mice (Fig 2D). However, for half of all outcome measures, the effect sizes varied across the 3 laboratories (i.e., nonoverlapping CI_95_ intervals between laboratories) with some laboratories detecting a significant strain effect and some not (e.g., “OF centre time”; Fig 2E). Most interestingly, completely contradicting conclusions were found regarding the number of “rearings” in the NC in both designs (Fig 2G), reflecting an example of severely hampered reproducibility. Whereas in Lab A, C57BL/6J mice reared less often than DBA/2N mice; in Lab C, C57BL/6J mice were characterised by higher numbers of “rearing” behaviour than DBA/2N. By contrast, Lab B found no difference between the 2 mouse strains. Thus, with respect to this outcome measure, we found 3 different conclusions (i.e., lower levels, higher levels, and no difference) about the strain effect in 3 different laboratories.

**Fig 3:**
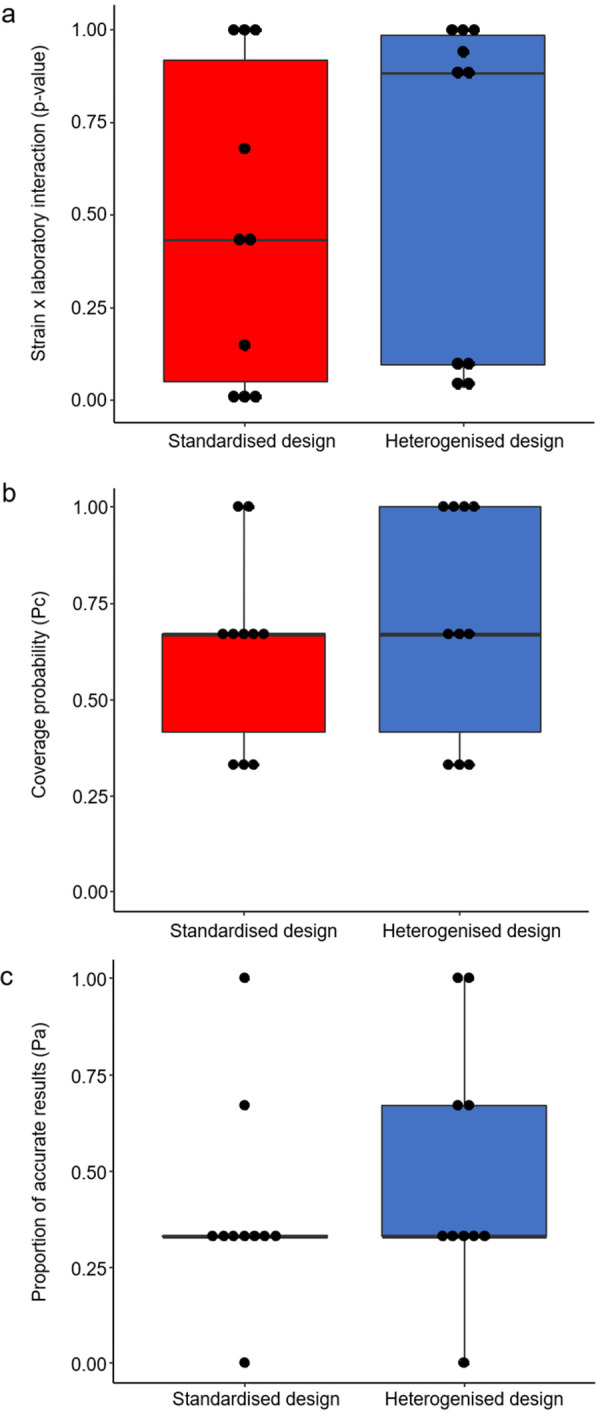
**Comparison of the reproducibility of the strain effect across different laboratories between a standardised (red) and heterogenised design (blue).** Shown are the following measurements of reproducibility: (a) Consistency of the strain effect across the 3 laboratories. This measurement is reflected by the *p*-value of the “strain-by-laboratory” interaction term of all 10 outcome measures. (b) Pc of all 10 outcome measures and (c) Pa of all 10 outcome measures. Data are presented as boxplots showing medians, 25% and 75% percentiles, and 5% and 95% percentiles. Black dots represent single values for each outcome measure in both designs. Statistics: Wilcoxon signed-rank test (paired, one-tailed, *n =* 10). The raw and processed data underlying this figure are available in the Figshare repositories https://figshare.com/s/f327175aa8b541ef01bd and https://figshare.com/s/2245cee43a544ee1ffff. Pa, proportion of accurate results; Pc, coverage probability.

To statistically compare the extent of reproducibility of the strain effect among the different laboratories between the standardised and the heterogenised design, 2 different approaches were used: First, we calculated the consistency of the strain effects across laboratories and second, we estimated how often (Pc) and how accurately (Pa) the results from each laboratory predicted the overall effect. Concerning the consistency of findings across laboratories, the *p*-values of the “strain-by-laboratory” interaction term did not differ significantly between the 2 designs (Fig 3A; Wilcoxon signed-rank test (paired, one-tailed, *n =* 10): V = 22, *p*-value = 0.31). Likewise, we could not detect any significant difference between the 2 designs in the performance to predict the overall effect (Fig 3B and 3C; Wilcoxon signed-rank test (paired, one-tailed, *n* = 10): Pc: V = 6, *p*-value = 0.39; Pa: V = 1.5, *p*-value = 0.14).

### Impact of experimenter allocation

The allocation of the experimenters to the experimental designs was randomly chosen. Thus, several different combinations of experimenters selected in each laboratory and allocated to each design are theoretically possible. To check whether alternative allocations of the experimenters would have altered the results when comparing both experimental designs, we repeated the analyses on basis of 10 additionally, randomly selected, alternative allocations (for details, see S3 Table).

Overall, in accordance with the initial finding, no significant differences could be detected regarding the reproducibility of the results between the experimental designs. More specifically, when examining the consistency of the strain effect of all 10 alternative allocations, one alternative allocation led to a significantly improved consistency of the strain effect in the heterogenised design (Wilcoxon signed-rank test (paired, one-tailed, *n =* 10): V = 9, *p*-value = 0.03). However, for the 9 remaining allocations, no significant differences between the experimental designs could be detected (Wilcoxon signed-rank test, paired, one-tailed, *n* = 10; for details, see S1 Fig and S6 Table). Likewise, with respect to the Pc and Pa ratios, no significant differences between both experimental designs could be found (Wilcoxon signed-rank test, paired, one-tailed, *n* = 10; for details, see S2 and S3 Figs and S6 Table).

### Components of variance

**Fig 4:**
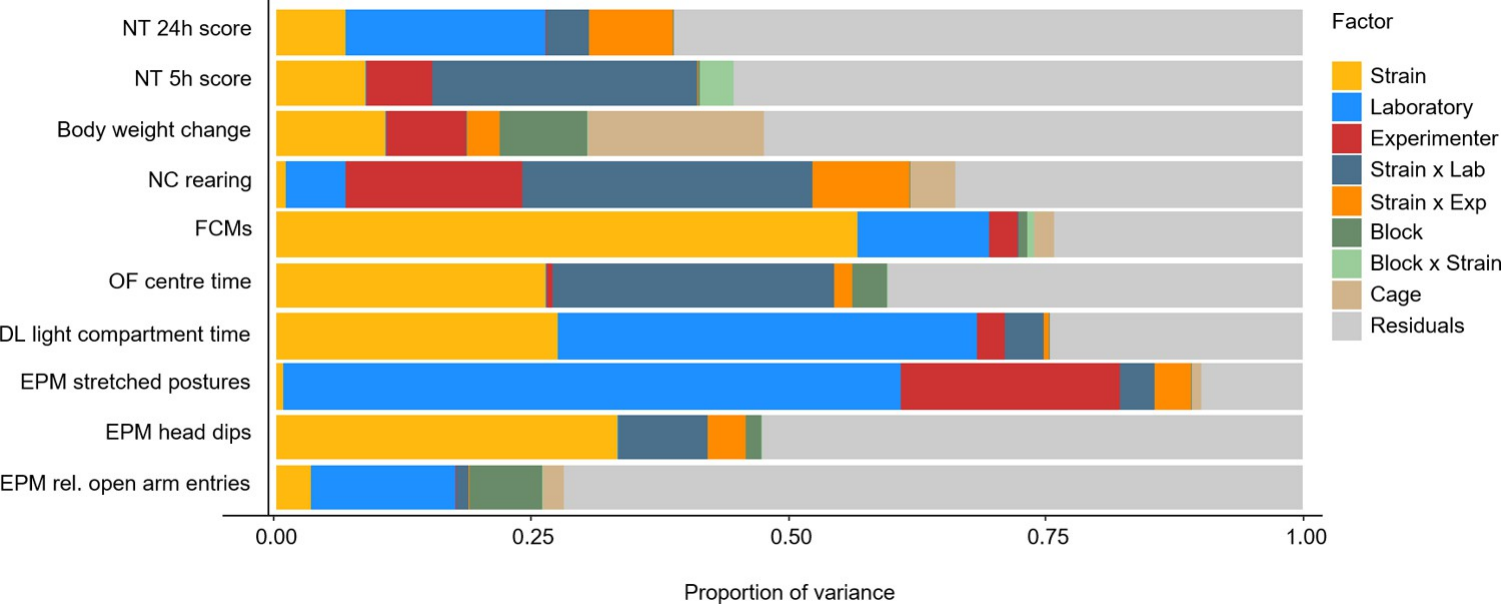
Proportion of variance explained by each factor. For each outcome measure, the total variance of the full dataset could be decomposed into the following sources using an LMM: between-strain variability (yellow), between-laboratory variability (blue), between-experimenter variability (red), strain-by-laboratory interaction variability (dark blue), strain-by-experimenter interaction variability (orange), between-block variability (dark green), strain-by-block interaction variability (light green), between-cage variability (beige), and between-individual variability (residuals, grey). Shown are point estimates of the proportion of variation explained by each factor. For details on 95% confidence intervals of these estimates, see S7 Table. The raw data underlying this figure are available in the Figshare repository https://figshare.com/s/f327175aa8b541ef01bd. DL, Dark Light; EPM, Elevated Plus Maze; FCMs, faecal corticosterone metabolites; LMM, linear mixed model; NC, Novel Cage; NT, Nest; OF, Open Field.

In addition, the full dataset was analysed to estimate the influence of the different factors on the variance of the data (for a summary, please see Fig 4). Although the specific amount of variation attributable to these different factors could only be estimated with some uncertainty, an overall pattern emerged. For 9 out of 10 outcome measures, the factor “laboratory” (main effect (blue) and interaction effect with strain (dark blue)) accounted for more variation than the factor “experimenter” (main effect (red) and interaction effect with strain (orange)). More precisely, the “laboratory” explained on average (median) 25% of the variation in the data (with a range from 0%, CI_95_ [0%, 17%] to 64%, CI_95_ [5%, 100%]), whereas on average, only 5% (median, with a range from 0%, CI_95_ [0%, 13%] to 27%, CI_95_ [2%, 97%]) of the total variance could be assigned to the factor “experimenter” (for details, please see S7 Table, which gives an overview of the variation explained by each factor including uncertainty measures (i.e., CI_95_ intervals) for all outcome measures). In addition, the proportion of variance that could not be assigned to any known source (= between-individual variability or residual variance, grey) accounted for 47% of the total variance (median, with a range from 10%, CI_95_ [8%, 13%] to 72%, CI_95_ [58%, 88%]). More specifically, in 6 out of 10 outcome measures, the residual variation represented the major source of variation (with a range from 41%, CI_95_ [34%, 49%] to 72%, CI_95_ [58%, 88%]).

## Discussion

The aim of this study was to empirically compare an experimenter heterogenisation strategy with a conventionally standardised design in a multilaboratory study. In contrast to our expectations, the experimenter heterogenisation led neither to more consistency across the 3 laboratories nor to a better performance in detecting the overall treatment effect compared to a conventionally standardised design. This finding was independent of the concrete constellation of experimenters contributing to the standardised and the heterogenised designs, as it remained the same when basing the analysis on 10 alternative, randomised allocations.

The reasons for these unexpected findings might be 2-fold: (1) either both experimental designs were characterised by “perfect” reproducibility across the 3 laboratories, creating a ceiling effect; or (2) the variation introduced by the different experimenters in the heterogenised design was not large enough to mimic the variation between laboratories.

Concerning the first point, the reproducibility of the results across the 3 laboratories was far from being “perfect”. Although, for some outcome measures, all 3 laboratories detected a significant strain effect of the same direction (e.g., “light compartment time in the DL” or “faecal corticosterone metabolites”); for half of all outcome measures, the conclusions from the single laboratories regarding the detected strain effect differed profoundly. The inconsistencies between laboratories ranged from variation in effect size to variation in the direction of the effects, including a case of significant effects in opposite directions (“rearings in the NC”).

Regarding the second point, although the experimenter identity is known as one of the top influencing factors in many fields of animal research [22,23,25], the systematic heterogenisation of this factor in the present study was not sufficient to improve the reproducibility of results across laboratories. Theoretically, the efficiency of a heterogenisation strategy is dependent on the amount of variation that is introduced by this specific factor. The experimenter heterogenisation in our study was realised on the basis of a multibatch design, meaning that each experimenter tested the animals in a different batch. This way, the introduced variation relied not only on the experimenter identities (e.g., sex, experience in handling mice), but it also comprised the environmental conditions under which the dedicated experimenter tested the animals (e.g., temperature, noise level). Including variation by splitting an experiment into several smaller ones spread over time (i.e., different batches) has already been shown to improve the reproducibility of results [34,36]. Hence, such an approach is particularly promising in exacerbating the experimenter effects. However, in our study, the factor “experimenter” accounted for on average only 5% of the observed variation in the data (median, with a range from 0%, CI_95_ [0%, 13%] to 27%, CI_95_ [2%, 97%]), indicating a rather small to moderate influence on outcome measures (for a detailed discussion on the experimenter effect in each laboratory, see S3 Text). Moreover, in 9 out of 10 outcome measures, the variation attributable to this factor appeared to be smaller than the variation introduced by the 3 laboratories. Although these explained variances are estimated with some uncertainty, it is likely that the variation introduced by multiple experimenters was therefore not sufficient enough to cover the variation that inevitably exists between different laboratories. Whether this finding is limited to the exemplarily chosen treatment effect in this study (i.e., physiological and behavioural mouse strain differences) or whether an experimenter heterogenisation might be more beneficial in studies involving extensive handling of the animals by the experimenter (cf. [23,27]) remains to be tested.

A glance at the growing body of heterogenisation literature demonstrates good evidence that heterogenisation strategies for single-laboratory studies in general help to render the results more generalisable and improve the reproducibility of the results across replicate experiments compared to standardised designs [30,31,33,34,36,37]. Interestingly, so far, this only holds true if the replication is done in the same laboratory, but not necessarily if the experiment is replicated at different locations [33]. Likewise, improved reproducibility was observed in a setting where environmental conditions between different laboratories did not differ largely [37]. Together, this argues for heterogenisation strategies in single-laboratory studies to have the potential to increase the inference space of the results and thereby to enhance the generalisability of the conclusions (i.e., across different experimenter identities or over time). At the same time, however, they may not broaden the inference space sufficiently to cover disparate laboratory environments and thus guarantee reproducibility across laboratories.

Recently, a database-driven simulation of a multilaboratory approach showed that a “laboratory heterogenisation”, including 2 to 4 laboratories in one study, rendered the results more robust and reproducible across replicates in different laboratories [32]. By using the laboratory as a heterogenisation factor, many different factors (e.g., light–dark cycle, housing conditions, strain of the animals) were systematically varied at the same time. Therefore, the laboratory served as kind of an “umbrella factor” for a set of known and unknown background factors, which was highly efficient in reflecting the “real-life” variation. However, the implementation of such a multilaboratory heterogenisation strategy in practice is highly demanding and might not be easily applicable to many studies.

Concerning sources of variation, the results of our study point to a further, so far widely overlooked aspect. Although the data were collected across different laboratories and experimenters, in 6 out of 10 outcome measures, our analysis suggests that a great proportion of variation (41%, CI_95_ [34%, 49%] to 72%, CI_95_ [58%, 88%] could not be explained by these factors or other often discussed sources of variance (e.g., cage). Instead, they were attributed to interindividual differences of unknown sources (residuals). Whether this observation only holds true for the here examined physiological and behavioural differences between inbred mouse strains or can be applied to other settings in animal research (e.g., to nonbehavioural readouts and interventions) needs to be further examined. Yet, our findings are in line with Kafkafi and colleagues [45], who also reported interindividual differences between mice to account for a high proportion of variation (up to 75%). Therefore, these findings may serve as an impressive example of how much biological variation can exist despite strict standardisation regimes. This is particularly alarming for all scientists conducting animal studies as standardisation is still considered as the “gold standard” to create controlled and homogeneous conditions in animal experiments and, thereby, to minimise random variation in the data (i.e., “noise”). It is likely that this high amount of unexplained variation emerges due to complex interactions between known but also unknown factors we are not even aware of [46]. Therefore, the identification of “umbrella factors” encompassing these known and unknown background factors might present a promising solution to integrate such uncontrolled variation systematically into the study design [38]. Thus, instead of trying to understand and eliminate all sources of unexplained variation in animal studies, future studies should aim at developing more efficient strategies to embrace such heterogeneity in study populations and, thereby, to improve generalisability and reproducibility of research findings.

## Supporting information

S1 Text Details on experimental procedures. (DOCX)

S2 Text Details on data analysis. (DOCX)

S3 Text Comparison of the results between different experimenters in each laboratory. (DOCX)

S1 Table Information about the characteristics of the environmental background and testing conditions for each laboratory. (XLSX)

S2 Table Information about the date of testing and characteristics of the experimenters in each laboratory. (XLSX)

S3 Table Allocations of the experimenters to the standardised and heterogenised design. Listed are the randomly chosen combination for the main analysis (Combination 0) and 10 alternative combinations. In each lab, the allocation process consisted of the following steps. First, all mice tested by one randomly chosen experimenter (Exp A—Exp D) were assigned to be part of the standardised design. Second, the remaining 3 experimenters in each lab were assigned to be part of the heterogenised design and one block of mice (B1 –B3) from each experimenter was pseudo-randomly selected for the heterogenised experiment in each lab. This was done in a way that in each heterogenised experiment, all 3 blocks were represented. Please note, due to the loss of animals, the datasets generated by Exp A and B in Lab C were only used for the heterogenised design in a restricted way. In detail, block B3 from Exp A and B2 from Exp B were excluded from the selection process.
(XLSX)

S4 Table Overview of data transformation for the main analysis (Comb 0) and the analysis of 10 alternative allocations of the experimenter to the experimental designs (Comb 1 –Comb 10). Transformations: log = log10 (y-1), sqrt = square root, asi*n =* arcsine (sqrt (y/100)).
(XLSX)

S5 Table Output of the LMM for both the standardised and heterogenised design. Presented are χ^2^- and *p*-values for the main effect of strain, laboratory, and the strain-by-laboratory interaction for all 10 selected outcome measures. Bold *p*-values indicate significant effects. Transformations (Transf): log = log10 (y-1), sqrt = square root. LMM, linear mixed model.
(XLSX)

S6 Table Overview of the statistical details of the Wilcoxon signed-rank test comparing standardised and heterogenised design. Presented are V- and *p*-values for the pairwise comparison of the strain-by-laboratory interaction, the Pc, and the Pa between both designs (*n* = 10, one-tailed). Bold *p*-values indicate significant differences between both designs. Shown is the output of the Wilcoxon signed-rank test for the main analysis (Comb 0) and for 10 alternative allocations of the experimenters to the designs (Comb 1 –Comb 10). Pa, proportion of accurate results; Pc, coverage probability.
(XLSX)

S7 Table Proportion of variance explained by each factor. Presented are point estimates of the component of variance analysis on the full data set for all 10 selected outcome measures. For the random factors in the LMM also confidence intervals (CI_95_) of the point estimates are presented in square brackets. Please note: confidence intervals were limited to the maximum possible range (i.e., [0,1]). LMM, linear mixed model.
(XLSX)

S8 Table Output of the LMM applied to the data of each laboratory, separately. Presented are F- and *p*-values for the main effect of strain, experimenter, and the strain-by-experimenter interaction for all 10 selected outcome measures. Bold *p*-values indicate significant effects. Transformations (Transf): log = log10 (y-1), sqrt = square root. LMM, linear mixed model.
(XLSX)

S1 Fig Consistency of the strain effect across laboratories of both the standardised (red) and the heterogenised (blue) design. Shown are *p*-values of the “strain-by-laboratory” interaction term across all 10 outcome measures for 10 alternative allocations of the experimenters to the designs (Combination 1–10). Data are presented as boxplots showing medians, 25% and 75% percentiles, and 5% and 95% percentiles. Black dots represent single *p*-values for each outcome measure in both designs. Statistics: Wilcoxon signed-rank test (paired, one-tailed, *n =* 10), *p ≤ 0.05. The raw and processed data underlying this figure are available in the Figshare repositories https://figshare.com/s/f327175aa8b541ef01bd and https://figshare.com/s/2245cee43a544ee1ffff.
(JPG)

S2 Fig Comparison of the Pc between both the standardised (red) and the heterogenised (blue) design. Shown are Pc ratios of 10 outcome measures for 10 alternative allocations of the experimenters to the designs (Combination 1–10). Data are presented as boxplots showing medians, 25% and 75% percentiles, and 5% and 95% percentiles. Black dots represent single values for each outcome measure in both designs. Statistics: Wilcoxon signed-rank test (paired, one-tailed, *n =* 10). The raw and processed data underlying this figure are available in the Figshare repositories https://figshare.com/s/f327175aa8b541ef01bd and https://figshare.com/s/2245cee43a544ee1ffff. Pc, coverage probability.
(JPG)

S3 Fig Comparison of the Pa between both the standardised (red) and the heterogenised (blue) design. Shown are Pa ratios of 10 outcome measures for 10 alternative allocations of the experimenters to the designs (Combination 1–10). Data are presented as boxplots showing medians, 25% and 75% percentiles, and 5% and 95% percentiles. Black dots represent single values for each outcome measure in both designs. Statistics: Wilcoxon signed-rank test (paired, one-tailed, *n =* 10). The raw and processed data underlying this figure are available in the Figshare repositories https://figshare.com/s/f327175aa8b541ef01bd and https://figshare.com/s/2245cee43a544ee1ffff. Pa, proportion of accurate results.
(JPG)

S4 Fig Frequency distribution of Spearman correlation coefficients between each pair of the 10 selected outcome measures (*n =* 45 pairs). The raw data underlying this figure are available in the Figshare repository https://figshare.com/s/f327175aa8b541ef01bd.
(JPG)

S5 Fig Relative open arm entries in the EPM shown by C57BL/6J (dark grey) and DBA/2N (light grey) mice in each laboratory. Results are displayed separately for each experimenter (Exp A–Exp D) conducting a full experiment (*n* = 12) in each laboratory. Data are presented as boxplots showing medians, 25% and 75% percentiles, and 5% and 95% percentiles. Statistics: LMMs followed by Tukey’s test for post hoc pairwise comparisons of the means. The analyses were conducted separately for the data of each laboratory, * *p* < 0.05. Abbreviations are indicating a significant strain effect (Strain*), experimenter effect (Exp*), or strain-by-experimenter interaction (Strain × Exp*). The raw data underlying this figure are available in the Figshare repository https://figshare.com/s/f327175aa8b541ef01bd. EPM, Elevated Plus Maze; LMM, linear mixed model.
(JPG)

S6 Fig Number of “head dips” in the EPM shown by C57BL/6J (dark grey) and DBA/2N (light grey) mice in each laboratory. Results are displayed separately for each experimenter (Exp A–Exp D) conducting a full experiment (*n =* 12) in each laboratory. Data are presented as boxplots showing medians, 25% and 75% percentiles, and 5% and 95% percentiles. Statistics: LMMs followed by Tukey’s test for post hoc pairwise comparisons of the means. The analyses were conducted separately for the data of each laboratory, * *p* < 0.05. Abbreviations are indicating a significant strain effect (Strain*), experimenter effect (Exp*), or strain-by-experimenter interaction (Strain × Exp*). The raw data underlying this figure are available in the Figshare repository https://figshare.com/s/f327175aa8b541ef01bd. EPM, Elevated Plus Maze; LMM, linear mixed model.
(JPG)

S7 Fig Number of “stretched postures” in the EPM shown by C57BL/6J (dark grey) and DBA/2N (light grey) mice in each laboratory. Results are displayed separately for each experimenter (Exp A–Exp D) conducting a full experiment (*n =* 12) in each laboratory. Data are presented as boxplots showing medians, 25% and 75% percentiles, and 5% and 95% percentiles. Statistics: LMMs followed by Tukey’s test for post hoc pairwise comparisons of the means. The analyses were conducted separately for the data of each laboratory, * *p* < 0.05. Abbreviations are indicating a significant strain effect (Strain*), experimenter effect (Exp*), or strain-by-experimenter interaction (Strain × Exp*). The raw data underlying this figure are available in the Figshare repository https://figshare.com/s/f327175aa8b541ef01bd. EPM, Elevated Plus Maze; LMM, linear mixed model.
(JPG)

S8 Fig Time spent in the light compartment in the DL test by C57BL/6J (dark grey) and DBA/2N (light grey) mice, respectively. Results are displayed separately for each experimenter (Exp A–Exp D) conducting a full experiment (*n =* 12) in each laboratory. Data are presented as boxplots showing medians, 25% and 75% percentiles, and 5% and 95% percentiles. Statistics: LMMs followed by Tukey’s test for post hoc pairwise comparisons of the means. The analyses were conducted separately for the data of each laboratory, * *p* < 0.05. Abbreviations are indicating a significant strain effect (Strain*), experimenter effect (Exp*), or strain-by-experimenter interaction (Strain × Exp*). The raw data underlying this figure are available in the Figshare repository https://figshare.com/s/f327175aa8b541ef01bd. DL, Dark Light; LMM, linear mixed model.
(JPG)

S9 Fig Time spent in the centre in the OF test by C57BL/6J (dark grey) and DBA/2N (light grey) mice, respectively. Results are displayed separately for each experimenter (Exp A–Exp D) conducting a full experiment (*n =* 12) in each laboratory. Data are presented as boxplots showing medians, 25% and 75% percentiles, and 5% and 95% percentiles. Statistics: LMMs followed by Tukey’s test for post hoc pairwise comparisons of the means. The analyses were conducted separately for the data of each laboratory, * *p* < 0.05. Abbreviations are indicating a significant strain effect (Strain*), experimenter effect (Exp*), or strain-by-experimenter interaction (Strain × Exp*). The raw data underlying this figure are available in the Figshare repository https://figshare.com/s/f327175aa8b541ef01bd. LMM, linear mixed model; OF, Open Field.
(JPG)

S10 Fig FCMs of C57BL/6J (dark grey) and DBA/2N (light grey) mice, respectively. Results are displayed separately for each experimenter (Exp A–Exp D) conducting a full experiment (*n =* 12) in each laboratory. Data are presented as boxplots showing medians, 25% and 75% percentiles, and 5% and 95% percentiles. Statistics: LMMs followed by Tukey’s test for post hoc pairwise comparisons of the means. The analyses were conducted separately for the data of each laboratory, * *p* < 0.05. Abbreviations are indicating a significant strain effect (Strain*), experimenter effect (Exp*), or strain-by-experimenter interaction (Strain × Exp*). The raw data underlying this figure are available in the Figshare repository https://figshare.com/s/f327175aa8b541ef01bd. FCMs, faecal corticosterone metabolites; LMM, linear mixed model.
(JPG)

S11 Fig Number of “rearings” in the NC test shown by C57BL/6J (dark grey) and DBA/2N (light grey) mice, respectively. Results are displayed separately for each experimenter (Exp A–Exp D) conducting a full experiment (*n =* 12) in each laboratory. Data are presented as boxplots showing medians, 25% and 75% percentiles, and 5% and 95% percentiles. Statistics: LMMs followed by Tukey’s test for post hoc pairwise comparisons of the means. The analyses were conducted separately for the data of each laboratory, * *p* < 0.05. Abbreviations are indicating a significant strain effect (Strain*), experimenter effect (Exp*), or strain-by-experimenter interaction (Strain × Exp*). The raw data underlying this figure are available in the Figshare repository https://figshare.com/s/f327175aa8b541ef01bd. LMM, linear mixed model; NC, Novel Cage.
(JPG)

S12 Fig Weight gain during the test phase of C57BL/6J (dark grey) and DBA/2N (light grey) mice, respectively. Results are displayed separately for each experimenter (Exp A–Exp D) conducting a full experiment (*n =* 12) in each laboratory. Data are presented as boxplots showing medians, 25% and 75% percentiles, and 5% and 95% percentiles. Statistics: LMMs followed by Tukey’s test for post hoc pairwise comparisons of the means. The analyses were conducted separately for the data of each laboratory, * *p* < 0.05. Abbreviations are indicating a significant strain effect (Strain*), experimenter effect (Exp*), or strain-by-experimenter interaction (Strain × Exp*). The raw data underlying this figure are available in the Figshare repository https://figshare.com/s/f327175aa8b541ef01bd. LMM, linear mixed model.
(JPG)

S13 Fig NT test score after 5 h for C57BL/6J (dark grey) and DBA/2N (light grey) mice, respectively. Results are displayed separately for each experimenter (Exp A–Exp D) conducting a full experiment (*n* = 12) in each laboratory. Data are presented as boxplots showing medians, 25% and 75% percentiles, and 5% and 95% percentiles. Statistics: LMMs followed by Tukey’s test for post hoc pairwise comparisons of the means. The analyses were conducted separately for the data of each laboratory, * *p* < 0.05. Abbreviations are indicating a significant strain effect (Strain*), experimenter effect (Exp*), or strain-by-experimenter interaction (Strain × Exp*). The raw data underlying this figure are available in the Figshare repository https://figshare.com/s/f327175aa8b541ef01bd. LMM, linear mixed model; NT, Nest.
(JPG)

S14 Fig NT test score after 24 h for C57BL/6J (dark grey) and DBA/2N (light grey) mice, respectively. Results are displayed separately for each experimenter (Exp A–Exp D) conducting a full experiment (*n* = 12) in each laboratory. Data are presented as boxplots showing medians, 25% and 75% percentiles, and 5% and 95% percentiles. Statistics: LMMs followed by Tukey’s test for post hoc pairwise comparisons of the means. The analyses were conducted separately for the data of each laboratory, * *p* < 0.05. Abbreviations are indicating a significant strain effect (Strain*), experimenter effect (Exp*), or strain-by-experimenter interaction (Strain × Exp*). The raw data underlying this figure are available in the Figshare repository https://figshare.com/s/f327175aa8b541ef01bd. LMM, linear mixed model; NT, Nest.
(JPG)

## Abbreviations

DL: Dark Light
EPM: Elevated Plus Maze
LMM: linear mixed model
NC: Novel Cage
NT: Nest
OF: Open Field
Pa: proportion of accurate results
Pc: coverage probability
PND: postnatal day

## References

[1] Begley CG, Ellis LM, NA, Begley CG, Ellis LM. Raise standards for preclinical cancer research. Nature. 2012;483:531–3. doi: 10.1038/483531a 22460880

[2] Open Science Collaboration. Estimating the reproducibility of psychological science. Science. 2015;349:aac4716. doi: 10.1126/science.aac4716 26315443

[3] Prinz F, Schlange T, Asadullah K. Believe it or not: How much can we rely on published data on potential drug targets? Nat Rev Drug Discov. 2011;10:712–3. doi: 10.1038/nrd3439-c1 21892149

[4] Nosek BA, Errington TM. Reproducibility in cancer biology: Making sense of replications. Elife. 2017;6:e23383. doi: 10.7554/eLife.23383 28100398 PMC5245957

[5] Baker M. 1,500 scientists lift the lid on reproducibility. Nature. 2016;533:452–4. doi: 10.1038/533452a 27225100

[6] Begley CG, Ioannidis JPA. Reproducibility in science: Improving the standard for basic and preclinical research. Circ Res. 2015;116:116–26. doi: 10.1161/CIRCRESAHA.114.303819 25552691

[7] Head ML, Holman L, Lanfear R, Kahn AT, Jennions MD. The extent and consequences of p-hacking in science. PLoS Biol. 2015;13:1–15. doi: 10.1371/journal.pbio.1002106 25768323 PMC4359000

[8] Kerr NL. HARKing: Hypothesizing after the results are known. Pers Soc Psychol Rev. 1998;2:196–217. doi: 10.1207/s15327957pspr0203_4 15647155

[9] Simmons JP, Nelson LD, Simonsohn U. False-positive psychology: Undisclosed flexibility in data collection and analysis allows presenting anything as significant. Psychol Sci. 2011;22:1359–66. doi: 10.1177/0956797611417632 22006061

[10] Fraser H, Parker T, Nakagawa S, Barnett A, Fidler F. Questionable research practices in ecology and evolution. PLoS ONE. 2018;13. doi: 10.1371/journal.pone.0200303 30011289 PMC6047784

[11] Nosek BA, Alter G, Banks GC, Borsboom D, Bowman SD, Breckler SJ, et al. Promoting an open research culture. Science. 2015;348:1422–5. doi: 10.1126/science.aab2374 26113702 PMC4550299

[12] Kilkenny C, Browne WJ, Cuthill IC, Emerson M, Altman DG. Improving bioscience research reporting: The arrive guidelines for reporting animal research. PLoS Biol. 2010;8. 10.1371/journal.pbio.1000412 PMC2893951 20613859

[13] Du Sert PN, Hurst V, Ahluwalia A, Alam S, Avey MT, Baker M, et al. The ARRIVE guidelines 2.0: Updated guidelines for reporting animal research*. J Cereb Blood Flow Metab. 2020;40:1769–77. doi: 10.1177/0271678X20943823 32663096 PMC7430098

[14] Smith AJ, Clutton RE, Lilley E, Hansen KEA, Brattelid T. PREPARE: guidelines for planning animal research and testing. Lab Anim. 2018;52:135–41. doi: 10.1177/0023677217724823 28771074 PMC5862319

[15] NPQIP Collaborative Group. Did a change in Nature journals’ editorial policy for life sciences research improve reporting? BMJ Open Sci. 2019;3. doi: 10.1136/bmjos-2017-000035 35047682 PMC8647608

[16] Pigliucci M. Phenotypic plasticity: beyond nature and nurture. JHU Press; 2001.

[17] Freund J, Brandmaier AM, Lewejohann L, Kirste I, Kritzler M, Krüger A, et al. Emergence of individuality in genetically identical mice. Science. 2013;340:756–9. doi: 10.1126/science.1235294 23661762

[18] Freund J, Brandmaier AM, Lewejohann L, Kirste I, Kritzler M, Krüger A, et al. Association between exploratory activity and social individuality in genetically identical mice living in the same enriched environment. Neuroscience. 2015;309:140–52. doi: 10.1016/j.neuroscience.2015.05.027 25987202

[19] Voelkl B, Altman NS, Forsman A, Forstmeier W, Gurevitch J, Jaric I, et al. Reproducibility of animal research in light of biological variation. Nat Rev Neurosci. 2020;21:384–93. doi: 10.1038/s41583-020-0313-3 32488205

[20] Crabbe JC, Wahlsten D, Dudek BC. Genetics of mouse behavior: interactions with laboratory environment. Science. 1999;284:1670–2. doi: 10.1126/science.284.5420.1670 10356397

[21] Wahlsten D, Metten P, Phillips TJ, Boehm 2nd SL, Burkhart-Kasch S, Dorow J, et al. Different data from different labs: Lessons from studies of gene-environment interaction. J Neurobiol. 2003;54:283–311. doi: 10.1002/neu.10173 12486710

[22] Sorge RE, Martin LJ, Isbester KA, Sotocinal SG, Rosen S, Tuttle AH, et al. Olfactory exposure to males, including men, causes stress and related analgesia in rodents. Nat Methods. 2014;11:629–32. doi: 10.1038/nmeth.2935 24776635

[23] Bohlen M, Hayes ER, Bohlen B, Bailoo JD, Crabbe JC, Wahlsten D. Experimenter effects on behavioral test scores of eight inbred mouse strains under the influence of ethanol. Behav Brain Res. 2014;272:46–54. doi: 10.1016/j.bbr.2014.06.017 24933191 PMC4968576

[24] López-Aumatell R, Martínez-Membrives E, Vicens-Costa E, Cañete T, Blázquez G, Mont-Cardona C, et al. Effects of environmental and physiological covariates on sex differences in unconditioned and conditioned anxiety and fear in a large sample of genetically heterogeneous (N/Nih-HS) rats. Behav Brain Funct. 2011;7:1–15. doi: 10.1186/1744-9081-7-1 22118015 PMC3254066

[25] Gouveia K, Hurst JL. Reducing mouse anxiety during handling: Effect of experience with handling tunnels. PLoS ONE. 2013;8:e66401. doi: 10.1371/journal.pone.0066401 23840458 PMC3688777

[26] Meijer MK, Spruijt BM, Van Zutphen LFM, Baumans V. Effect of restraint and injection methods on heart rate and body temperature in mice. Lab Anim. 2006;40:382–91. doi: 10.1258/002367706778476370 17018209

[27] Chesler EJ, Wilson SG, Lariviere WR, Rodriguez-Zas SL, Mogil JS. Identification and ranking of genetic and laboratory environment factors influencing a behavioral trait, thermal nociception, via computational analysis of a large data archive. Neurosci Biobehav Rev. 2002;26:907–23. doi: 10.1016/s0149-7634(02)00103-3 12667496

[28] Richter SH, Garner JP, Würbel H. Environmental standardization: Cure or cause of poor reproducibility in animal experiments? Nat Methods. 2009;6:257–61. doi: 10.1038/nmeth.1312 19333241

[29] Richter SH. Automated home-cage testing as a tool to improve reproducibility of behavioral research? Front Neurosci. 2020;14:10–4. doi: 10.3389/fnins.2020.00010 32390795 PMC7193758

[30] Richter SH, Garner JP, Auer C, Kunert J, Würbel H. Systematic variation improves reproducibility of animal experiments. Nat Methods. 2010;7:167–8. doi: 10.1038/nmeth0310-167 20195246

[31] Bodden C, von Kortzfleisch VT, Karwinkel F, Kaiser S, Sachser N, Richter SH. Heterogenising study samples across testing time improves reproducibility of behavioural data. Sci Rep. 2019;9:1–9. doi: 10.1038/s41598-018-37186-2 31160667 PMC6547843

[32] Voelkl B, Vogt L, Sena ES, Würbel H. Reproducibility of preclinical animal research improves with heterogeneity of study samples. PLoS Biol. 2018;16:1–13. doi: 10.1371/journal.pbio.2003693 29470495 PMC5823461

[33] Richter SH, Garner JP, Zipser B, Lewejohann L, Sachser N, Touma C, et al. Effect of population heterogenization on the reproducibility of mouse behavior: A multi-laboratory study. PLoS ONE. 2011;6(1):e16461. doi: 10.1371/journal.pone.0016461 21305027 PMC3031565

[34] Karp NA, Wilson Z, Stalker E, Mooney L, Lazic SE, Zhang B, et al. A multi-batch design to deliver robust estimates of efficacy and reduce animal use–a syngeneic tumour case study. Sci Rep. 2020;10:1–10. doi: 10.1038/s41598-019-56847-4 32277094 PMC7148295

[35] Usui T, Macleod MR, McCann SK, Senior AM, Nakagawa S. Embrace heterogeneity to improve reproducibility: A perspective from meta-analysis of variation in preclinical research. bioRxiv. 2020. doi: 10.1101/2020.10.26.354274

[36] von Kortzfleisch VT, Karp NA, Palme R, Kaiser S, Sachser N, Richter SH. Improving reproducibility in animal research by splitting the study population into several ‘mini-experiments’. Sci Rep. 2020;10:16579. doi: 10.1038/s41598-020-73503-4 33024165 PMC7538440

[37] Milcu A, Puga-Freitas R, Ellison AM, Blouin M, Scheu S, Freschet GT, et al. Genotypic variability enhances the reproducibility of an ecological study. Nat Ecol Evol. 2018;2:279–87. doi: 10.1038/s41559-017-0434-x 29335575

[38] Richter SH, von Kortzfleisch V. It is time for an empirically informed paradigm shift in animal research. Nat Rev Neurosci. 2020;21:660. doi: 10.1038/s41583-020-0369-0 32826977

[39] Kappel S, Hawkins P, Mendl MT. To group or not to group? Good practice for housing male laboratory mice. Animals. 2017;7(12):88. 10.3390/ani7120088 PMC5742782 29186765

[40] Melotti L, Kästner N, Eick AK, Schnelle AL, Palme R, Sachser N, et al. Can live with ‘em, can live without ‘em: Pair housed male C57BL/6J mice show low aggression and increasing sociopositive interactions with age, but can adapt to single housing if separated. Appl Anim Behav Sci. 2019;214:79–88.

[41] Urbaniak GC, Plous S. Research Randomizer (Version 4.0). 2013. Available from: http://www.randomizer.org/

[42] Lad HV, Liu L, Paya-Cano JL, Parsons MJ, Kember R, Fernandes C, et al. Behavioural battery testing: Evaluation and behavioural outcomes in 8 inbred mouse strains. Physiol Behav. 2010;99:301–16. doi: 10.1016/j.physbeh.2009.11.007 19931548

[43] R Core Team. R: A language and environment for statistical computing. Vienna, Austria: R Foundation for Statistical Computing; 2017. Available from: https://www.R-project.org/.

[44] Faul F, Erdfelder E, Buchner A, Lang A-G. Statistical power analyses using G*Power 3.1: Tests for correlation and regression analyses. Behav Res Methods. 2009;41:1149–60. doi: 10.3758/BRM.41.4.1149 19897823

[45] Kafkafi N, Benjamini Y, Sakov A, Elmer GI, Golani I. Genotype-environment interactions in mouse behavior: A way out of the problem. PNAS. 2005;102(12):4619–24. doi: 10.1073/pnas.0409554102 15764701 PMC555503

[46] Mogil JS. Laboratory environmental factors and pain behavior: The relevance of unknown unknowns to reproducibility and translation. Lab Anim (NY). 2017;46:136–41. doi: 10.1038/laban.1223 28328894

