# Correction: Do multiple experimenters improve the reproducibility of animal studies?

**DOI:** 10.1371/journal.pbio.3001948

**Published:** 2022-12-20

**Authors:** Vanessa Tabea von Kortzfleisch, Oliver Ambrée, Natasha A. Karp, Neele Meyer, Janja Novak, Rupert Palme, Marianna Rosso, Chadi Touma, Hanno Würbel, Sylvia Kaiser, Norbert Sachser, S. Helene Richter

The Funding statement is incomplete. The correct Funding statement is:

The overall project was supported by a grant from the German Research Foundation (DFG) to S.H.R. (RI 2488/3 1). Project parts conducted within the laboratory of H.W. in Switzerland were additionally supported by a grant from the Swiss National Science Foundation to H.W. (SNF Grant No. 310030–179254). The funders had no role in study design, data collection and analysis, decision to publish, or preparation of the manuscript.
