# Supplementary material for: Do multiple experimenters improve the reproducibility of animal studies?
Source: PLoS Biol. 2022 May 5;20(5):e3001564. doi: 10.1371/journal.pbio.3001564 (PMC9070896; doi:10.1371/journal.pbio.3001564)
Supplement: S1 Text — (DOCX) [file pbio.3001564.s001.docx]

**S1 Text: Details on experimental procedures**

**Elevated Plus Maze test**. The EPM is a well-established test to examine the exploratory locomotion and anxiety-like behaviour of an animal (Lister 1987). The EPM apparatus consisted of a plus-shaped platform, elevated above the ground and was composed of two opposing open and two opposing closed arms which were connected via a central square. The closed arms were surrounded by walls (for details on the apparatus measures and lighting conditions in each laboratory see Supplementary S1 Table). After spending 1 min in an empty transport box, the animal was placed into the centre zone facing a closed arm. The behaviour of the animal in the EPM was recorded for 5 min by a camera from above and automatically analysed by a video tracking software (ANY-maze, Stoelting Co. or Ethovision, Noldus, see Supplementary S1 Table). Outcome measures taken were the total distance travelled, the distance travelled on the open arms, the relative amount of entries into and the relative time spent in the open arms. In addition, the number of ‘head dips’ (‘mouse lowers its head over the side of an open arm with its ears protruding over the edge’; cf. Bodden at al. 2019) and the amount of ‘stretched postures’ (‘mouse elongates the body, while the hind paws maintain fixed followed by subsequent retraction of the body’) were counted manually by the experimenter from the video recordings. Due to technical reasons, the data from two mice had to be excluded for the EPM.

**Dark Light test**. The DL is a paradigm to examine anxiety-like behaviour and exploratory locomotion (Crawley & Goodwin 1980). The DL apparatus consisted of a box divided into a dark compartment (one third of the space) and a light compartment. Both compartments were connected via a sliding door (for details on the apparatus measures and lighting conditions in each laboratory see Supplementary S1 Table). In the beginning of the DL, the animal was placed for 1 min in the dark compartment. After this time, the sliding door was opened, and the animal was allowed to freely explore the apparatus for 5 min. During that time, the behaviour of the mouse was recorded by a camera from above and automatically analysed by a video tracking software (ANY-maze, Stoelting Co. or Ethovision, Noldus, see Supplementary S1 Table). Outcome measures were the latency until the first entry into the light compartment, the total number of entries into and the time spent in the light compartment. Due to technical issues, data from one mouse had to be excluded for the DL.

**Open Field test**. Similar to the EPM and the DL, the OF is a paradigm to assess the exploratory locomotion and anxiety-like behaviour of mice (Crawley 1985). The apparatus consisted of an open arena surrounded by walls, which was enlightened from above. The area of this arena was divided into a wall and a centre zone (for details on the apparatus measures and lighting conditions in each laboratory see Supplementary S1 Table). After spending 1 min in an empty transport box, the animal was placed in the OF facing the wall and was allowed to freely explore the arena for 5 min. During that time, the animal was recorded by a camera. The number of entries into the centre zone, the distance travelled, and the time spent in the centre zone as well as the total distance travelled in the OF was automatically analysed by a video tracking software (ANY-maze, Stoelting Co. or Ethovision, Noldus). In addition, the number of faecal boli in the OF was counted by the experimenter at the end of the test.

**Novel Cage test**. The NC is a paradigm to observe exploratory locomotion in a new environment by resembling a cage cleaning routine (Fuss et al. 2013). A new standard housing cage was filled with fresh bedding material (for details see Supplementary S1 Table). Mice were placed into this new housing cage for a duration of 5 min and the frequency of ‘rearing’ (‘a mouse raises itself on its hindpaws and stretches its snout into the air’) was manually recorded by the experimenter as a measure of vertical exploration. Due to technical reasons, the data of two mice is missing for the NC.

**Nest test**. To assess the nest building ability of the animals, one hour prior to the onset of the dark phase, shelters and nesting material (if present) were removed from the cages for 24 h and a cotton nestlet (NES3600, Ancare) was provided. The quality of the nests was scored after 5 and 24 h for each cage (one score per cage). The definition of scores was adopted from Deacon (2006) and ranged from 1 to 5.

**Faecal corticosterone metabolites**. To determine stress hormone levels non-invasively, faecal corticosterone metabolites (FCMs) were measured. Therefore, on PND 73 ± 2 mice were transferred one hour after the onset of the dark phase into new housing cages to collect faecal samples. After a 3 h collecting phase, all defecated faecal boli were sampled individually for each animal and frozen at −20 °C. Due to technical reasons, the sample of one mouse could not be collected. Samples were dried and homogenised and an aliquot of 0.05 g each was extracted with 1 ml of 80% methanol. Whereas the faecal samples were collected by each experimenter, the extraction procedure was done by one person per laboratory. Finally, the samples from all laboratories were sent to the University of Veterinary Medicine in Vienna to determine FCMs. For this a 5α-pregnane-3β,11β,21-triol-20-one enzyme immunoassay was used which was established and successfully validated to measure FCMs in mice (for details see Touma et al. 2003, 2004). Intra- and inter-assay coefficients of variation were below 10%.

References:

Bodden, C. et al. Heterogenising study samples across testing time improves reproducibility of behavioural data. Sci. Rep. 9, 1–9 (2019). doi:10.1038/s41598-019-44705-2

Crawley, J. N. Exploratory behavior models of anxiety in mice. Neurosci. & Biobehav. Rev. 9, 37–44 (1985).

Crawley, J. & Goodwin, F. K. Preliminary report of a simple animal behavior model for the anxiolytic effects of benzodiazepines. Pharmacol. Biochem. Behav. 13, 167–170 (1980).

Deacon, R. M. J. Assessing nest building in mice. Nat. Protoc. 1, 1117–1119 (2006).

Fuss, J. et al. Are you real? Visual simulation of social housing by mirror image stimulation in single housed mice. Behav. Brain Res. 243, 191–198 (2013).

Lister, R. G. The use of a plus-maze to measure anxiety in the mouse. Psychopharmacology (Berl). 92, 180–185 (1987).

Touma, C., Sachser, N., Möstl, E. & Palme, R. Effects of sex and time of day on metabolism and excretion of corticosterone in urine and feces of mice. Gen. Comp. Endocrinol. 130, 267–278 (2003).

Touma, C., Palme, R. & Sachser, N. Analyzing corticosterone metabolites in fecal samples of mice: A noninvasive technique to monitor stress hormones. Horm. Behav. 45, 10–22 (2004).
