# Supplementary material for: Do multiple experimenters improve the reproducibility of animal studies?
Source: PLoS Biol. 2022 May 5;20(5):e3001564. doi: 10.1371/journal.pbio.3001564 (PMC9070896; doi:10.1371/journal.pbio.3001564)
Supplement: S2 Text — (DOCX) [file pbio.3001564.s002.docx]

**S2 Text: Details on data analysis**

**Reproducibility of the strain effect across laboratories**

I.) Consistency of the strain effect across laboratories

The following linear mixed model (LMM, equation 1) was applied to both designs (standardised and heterogenised) to assess the consistency of the strain effect across laboratories (‘strain-by-laboratory’-interaction) as a measurement of reproducibility.

Model written in formal terms:

*y_ijmk_* = *µ* + *a_i_* + *b_j_* + c_ij_ + *d_m_* + *f_im_* + *ε_ijmk_* Equation (1)

where *i* = 1, …, *n_S_*, *j* = 1, …, *n_R_*, *m* = 1,…, *n_B_* and *k* = 1,…, *n_ijm_*. *a_i_* indicates the main effect of the *i*th level of strain (treatment); *b_j_* represents laboratory as a random effect where *b_j_* ~ N(0,σ_b_^2^); *c_ij_* represents strain-by-laboratory-interaction as random effect where *c_ij_* ~ N(0,σ_c_^2^); *d_m_* represents block as a random effect nested in laboratory where *d_m_* ~ N(0,σ_d_^2^); *f_im_* represents strain-by-block-interaction as a random effect where *f_im_* ~ N(0,σ_f_^2^) and the error term *ε_ijmk_* ∼ N(0,σ_e_^2^).

Equation 1 written in layperson terms:

y = ‘strain’ + ‘laboratory’ + ‘strain x laboratory’ + ‘block’ + ‘block x strain’ Equation (1)

where ‘strain’ was included as fixed factor and ‘laboratory’, ‘strain-by-laboratory’-interaction, ‘block’ and ‘block-by-strain’-interaction as random factors. Moreover, the factor ‘block’ was nested within ‘laboratory’. The blocking factor was included in accordance with the randomised block design used, in which mice sharing the same micro-environment were treated as one ‘block’ (i.e. same rack position, see Fig 2). To check for the assumptions of parametric analysis, residuals were graphically examined for normal distribution, homoscedasticity, and the Shapiro-Wilk test was applied. When necessary, raw data were transformed using square root, logarithmic or arcsine transformations (see Supplementary S4 Table).

Typically, the contribution of a fixed effect to a model is assessed by examining the F-values as they return the relative variance that is explained by the term against the total variance of the data. For a random effect, however, the F-values cannot be determined. As the ‘strain-by-laboratory’-interaction constituted a random effect in the model, the F-values could not be assessed and p-values were used as a quantitative measurement for the contribution of this effect to the model (i.e., as a proxy for the F-values). The p-values are a function of the chi-square value of a Likelihood Ratio test assessing the random effect by comparing a full linear model versus a restricted linear model (i.e., without the interaction effect) and the degrees of freedom in the model. The degrees of freedom can be assumed to be the same in the analysis of both designs, since in both experimental designs the same sample size was used and the models have the same structure regarding the applied factor levels. Higher p-values of the interaction term indicate less impact of the laboratory on the strain effect and therefore, more consistency of the strain effect among laboratories and thus better reproducibility. Subsequently, the p-values of the ‘strain-by-laboratory’-interaction term of all 10 outcome measures were compared between the standardised and heterogenised design. This was done by using the Wilcoxon signed-rank test (paired, one-tailed). The Wilcoxon signed-rank test was chosen as a non-parametric test for dependent groups to provide a direct comparison of the reproducibility of the results between both designs using the same outcome measure (cf. von Kortzfleisch et al. 2020).

II.) Estimation of how often and how accurately the overall effect is predicted in the laboratories.

The performance of each experimental design to predict the overall effect size was assessed by the coverage probability (Pc) and the proportion of accurate results (Pa).

As a first step, the overall effect size of each outcome measure was estimated by conducting a random-effect meta-analysis on the data of all three laboratories independent of the experimental design. In detail, individual strain effect sizes and corresponding standard errors were calculated by applying the following linear mixed model (equation 2) to the data of each laboratory and experimental design, separately.

Written in formal terms:

*y_imk_* = *µ* + *a_i_* + *d_m_* + *f_im_* + *ε_imk_* Equation (2)

where *i* = 1, …, *n_S_*, *m* = 1,…, *n_B_* and *k* = 1,…, *n_ijm_*. *a_i_* indicates the main effect of the *i*th level of strain (treatment); *d_m_* represents block as a random effect *d_m_* ~ N(0,σ_d_^2^); *f_im_* represents strain-by-block-interaction as a random effect *f_im_* ~ N(0,σ_f_^2^) and the error term *ε_imk_* ∼ N(0,σ_e_^2^).

Written in layperson terms:

y = ‘strain’ + ‘block’ + ‘block x strain’ Equation (2)

where ‘strain’ was included as fixed effect and ‘block’ and the ‘block-by-strain’-interaction as random factors to account for the structure of the randomised block design in each laboratory (for details see Fig 2)

The random-effect meta-analysis was based on the individual strain effect sizes and standard errors of all laboratories deriving from equation 2. It was conducted using the R-package ‘metafor’ (Viechtbauer 2010, Version 2.1.0). The intercept of the following linear mixed model (equation 3) was used to return the overall effect sizes and corresponding CI_95_ of each outcome measure.

Written in formal terms:

*S_i_* = *µ* + *f_i_* + *ε_i_* Equation (3)

where *i* = 1, …, *n_R_*. *S_i_* represents the estimated strain effect sizes. *f_i_* indicates laboratory as a random effect and the error term *ε_i_* ∼ N(0,σ_e_^2^).

Written in layperson terms:

y = ‘laboratory’ Equation (3)

where ‘laboratory’ was included as random effect to account for the variation between laboratories.

Following this step, mean strain differences and CI_95_ were computed based on the LMM in equation 2 using the R-package ‘lsmeans’ (Lenth & Lenth 2018, Version 2.30.0) for each laboratory and both designs, separately. Then the Pc and Pa were assessed for each experimental design. The Pc was calculated by counting how often the CI_95_ of the different laboratories covered the overall effect size, whereas the Pa was determined by counting how often the laboratories predicted the overall effect accurately concerning its statistical significance. For the latter, it was examined whether the CI_95_ of the overall pooled effect overlapped with 0 (i.e., overall not significant effect) or not (i.e. overall significant effect). In detail, two requirements had to be met for a laboratory to be counted as predicting the overall effect accurately. The CI_95_ of the results from one laboratory had to include the overall effect and if CI_95_ of the overall effect included 0, then the CI_95_ of the laboratory also had to include 0 (for a graphical explanation of this concept see Voelkl et al. 2018 and von Kortzfleisch et al. 2020).

Finally, an analysis similar to the p-values of the interaction-term was conducted; the Pc and Pa ratios of all 10 outcome measures were compared between both designs using the Wilcoxon signed-rank test (paired, one-tailed).

**Components of variance**

To disentangle the different sources of variation and estimate the proportion of variance attributable to these factors, the following linear mixed model (LMM, equation 4) was applied to the full data set.

Written in formal terms:

*y_ijmk_* = *µ* + *a_i_* + *b_j_* + c_ij_ + *d_m_* + *f_im_* + *g_n_* + *h_in_* + *k_p_* + *ε_ijmr_* Equation (4)

where *i* = 1, …, *n_S_*, *j* = 1, …, *n_R_*, *m* = 1,…, *n_B_* and *k* = 1,…, *n_ijm_*. *a_i_* indicates the main effect of the *i*th level of strain (treatment); *b_j_* represents laboratory as a random effect where *b_j_* ~ N(0,σ_b_^2^); *c_ij_* represents strain-by-laboratory-interaction as random effect where *c_ij_* ~ N(0,σ_c_^2^); *d_m_* represents experimenter as a random effect nested in laboratory where *d_m_* ~ N(0,σ_d_^2^); *f_im_* represents strain-by-experimenter-interaction as a random effect where *f_im_* ~ N(0,σ_f_^2^); *g_n_* represents block as a random effect nested in laboratory and experimenter where *g_n_* ~ N(0,σ_d_^2^); *h_in_* represents strain-by-block-interaction as a random effect where *h_in_* ~ N(0,σ_f_^2^); *k_p_* represents cage as a random effect where *k_p_* ~ N(0,σ_f_^2^) and the error term *ε_ijmr_* ∼ N(0,σ_e_^2^).

Written in layperson terms:

y = ‘strain’ + ‘laboratory’ + ‘experimenter’ + ‘strain x laboratory’ + ‘strain x experimenter’ + ‘block’ + ‘block x strain’ + ‘cage’ Equation (4)

where ‘strain’ was included as fixed factor and ‘laboratory’, ‘experimenter’, ‘strain-by-laboratory’-interaction, ‘strain-by-experimenter’-interaction, ‘block’, ‘block-by-strain’-interaction and ‘cage’ as random factors. The factor ‘block’ was nested within ‘experimenter’ which was nested within ‘laboratory’. For the two outcome measures Nest test 5h score and Nest test 24h score, the random factor ‘cage’ was not included in the LMM, because scores were assessed at the cage level. For the component of variance analysis, the amount of estimated variance for each component in the LMM was calculated and divided by the total variation of the outcome measure to provide the relative explained variation by each factor.

References:

Lenth, R. & Lenth, M. R. Package ‘lsmeans’. Am. Stat. 34, 216–221 (2018).

Viechtbauer, W. Conducting meta-analyses in R with the metafor package. J. Stat. Softw. 36, 1–48 (2010).

Voelkl, B., Vogt, L., Sena, E. S. & Würbel, H. Reproducibility of preclinical animal research improves with heterogeneity of study samples. PLoS Biol. 16, 1–13 (2018).
doi:10.1371/journal.pbio.2003693

von Kortzfleisch, V. T. et al. Improving reproducibility in animal research by splitting the study population into several ‘mini-experiments’. Sci. Rep. 10, 16579 (2020).
