# Supplementary material for: Do multiple experimenters improve the reproducibility of animal studies?
Source: PLoS Biol. 2022 May 5;20(5):e3001564. doi: 10.1371/journal.pbio.3001564 (PMC9070896; doi:10.1371/journal.pbio.3001564)
Supplement: S3 Text — (DOCX) [file pbio.3001564.s003.docx]

**S3 Text: Comparison of the results between different experimenters in each laboratory**

In each laboratory, each experimenter conducted a ‘full’ experiment testing 12 mice per strain. This data was used in an additional analysis to examine the effect of the experimenter on the outcome measures within each laboratory. For this analysis, the following linear mixed model (equation 5) was applied to the data of each laboratory separately.

Written in layperson terms: Equation (5)

y = ‘strain’ + ‘experimenter’ + ‘strain x experimenter’ + ‘block’ + ‘block x strain’

where ‘strain’, ‘experimenter’ and ‘strain-by-laboratory’-interaction was included as fixed factors and ‘block’ and ‘block-by-strain’-interaction as random factors.

The comparison revealed that in some outcome measures (e.g. ‘Novel Cage rearing’, S11 Fig) the results from the two inbred mouse strains were significantly different between the different experimenters within one laboratory (for an overview of the statistical details see S8 Table). The experimenter affected the results either in an additive way (e.g. ‘Dark Light compartment time’ in Lab A, S8 Fig) or in a more complex, interactive way (e.g. ‘Novel Cage rearing’, S11 Fig). In the latter, some experimenters within one laboratory found a significant difference between the two strains of mice (e.g. Exp A and C in Lab C), whereas the results of the other two experimenters (Exp B and D) did not reveal a significant difference between the two strains. This is an impressive example that the reproducibility of results may not only be affected by different laboratory environments as seen in the data from the main analysis but can also be affected by the specific experimenter conducting the tests (Bohlen et al. 2014, Sorge et al. 2014). However, for other outcome measures (e.g. ‘head dips in the Elevated Plus Maze’, S6 Fig and ‘Open Field centre time’, S9 Fig) the results did not differ significantly between the different experimenters within one laboratory. Overall, significant interactions between the experimenter and the strain were solely found in outcome measures that were manually scored by the experimenter, such as the ‘number of rearings in the Novel Cage test’ and the ‘number of stretched postures in the Elevated Plus Maze’. In fact, this finding may indicate that manually scored outcome measures are more prone to be influenced by the experimenter identity than automatically tracked outcome measures (e.g. ‘Open Field centre time’). However, future studies are needed to approach this hypothesis more systematically by assessing the same outcome measures either manually or automatically.

References:

Bohlen, M., Hayes, E. R., Bohlen, B., Bailoo, J. D., Crabbe, J. C., & Wahlsten, D. Experimenter effects on behavioral test scores of eight inbred mouse strains under the influence of ethanol. Behavioural brain research, 272, 46-54 (2014).

Sorge, R. E. et al. Olfactory exposure to males, including men, causes stress and related analgesia in rodents. Nature methods, 11(6), 629-632 (2014).
