# Supplementary figures and images for: Do multiple experimenters improve the reproducibility of animal studies?
Source: PLoS Biol. 2022 May 5;20(5):e3001564. doi: 10.1371/journal.pbio.3001564 (PMC9070896; doi:10.1371/journal.pbio.3001564)

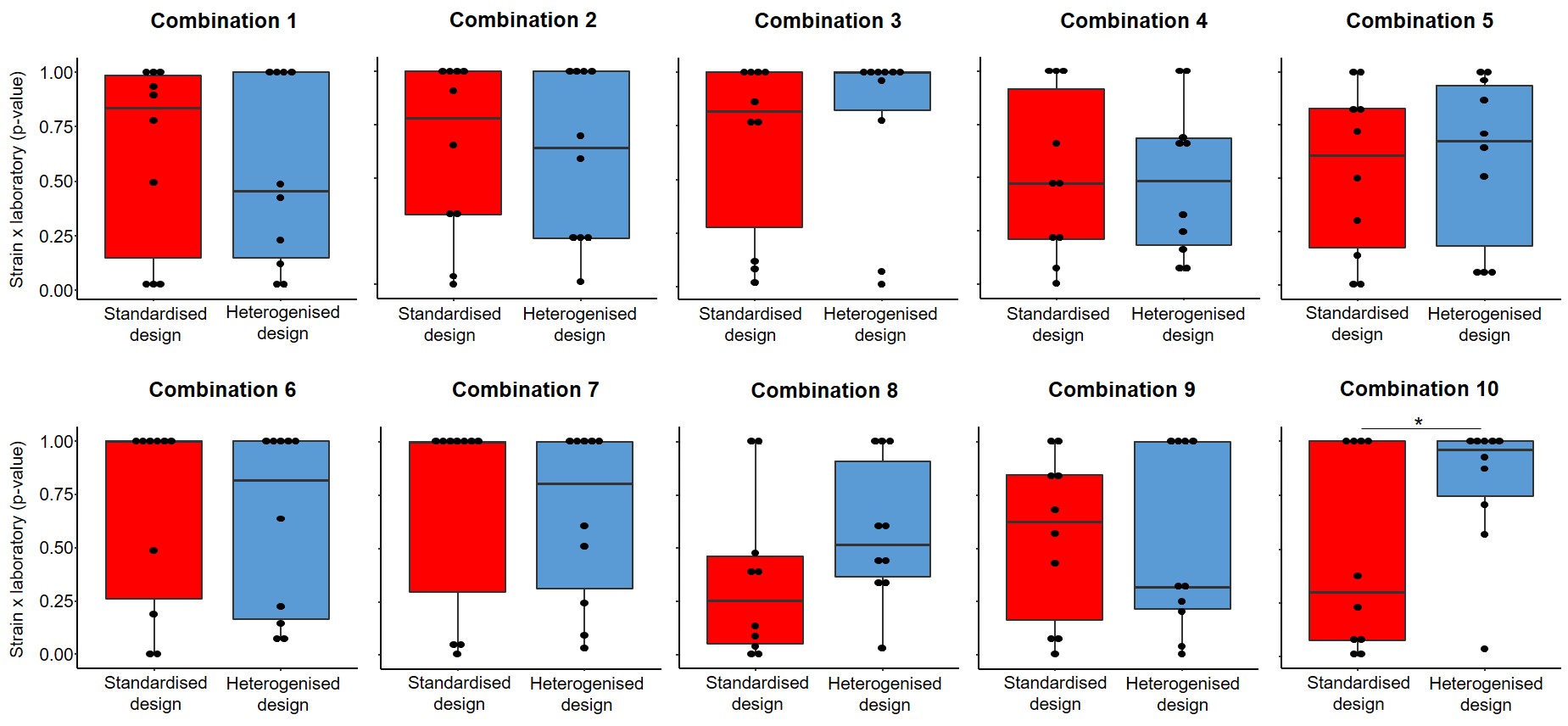

Supplement: S1 Fig — Shown are p-values of the “strain-by-laboratory” interaction term across all 10 outcome measures for 10 alternative allocations of the experimenters to the designs (Combination 1–10). Data are presented as boxplots showing medians, 25% and 75% percentiles, and 5% and 95% percentiles. Black dots represent single p-values for each outcome measure in both designs. Statistics: Wilcoxon signed-rank test (paired, one-tailed, n = 10), *p ≤ 0.05. The raw and processed data underlying this figure are available in the Figshare repositories https://figshare.com/s/f327175aa8b541ef01bd and https://figshare.com/s/2245cee43a544ee1ffff. (JPG) [file pbio.3001564.s012.jpg]

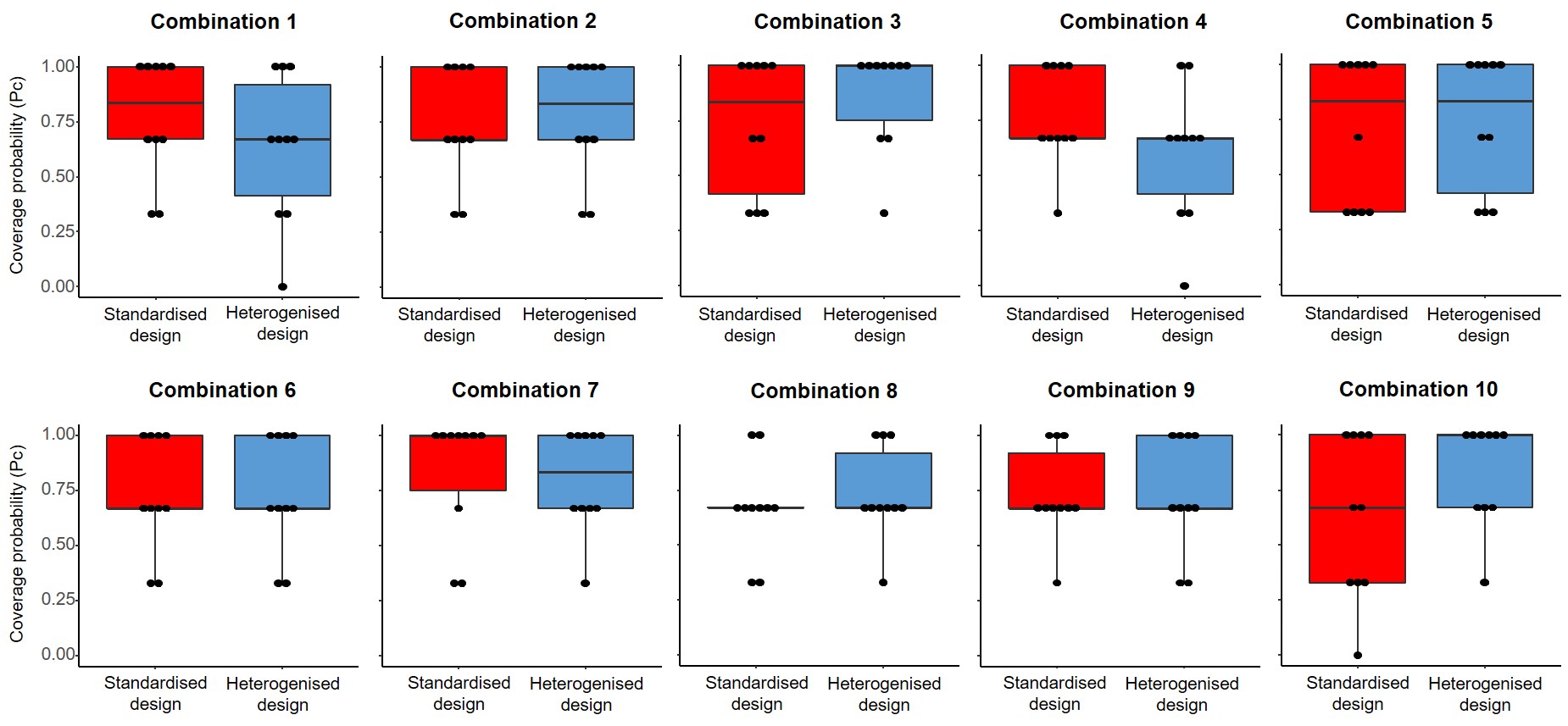

Supplement: S2 Fig — Shown are Pc ratios of 10 outcome measures for 10 alternative allocations of the experimenters to the designs (Combination 1–10). Data are presented as boxplots showing medians, 25% and 75% percentiles, and 5% and 95% percentiles. Black dots represent single values for each outcome measure in both designs. Statistics: Wilcoxon signed-rank test (paired, one-tailed, n = 10). The raw and processed data underlying this figure are available in the Figshare repositories https://figshare.com/s/f327175aa8b541ef01bd and https://figshare.com/s/2245cee43a544ee1ffff. Pc, coverage probability. (JPG) [file pbio.3001564.s013.jpg]

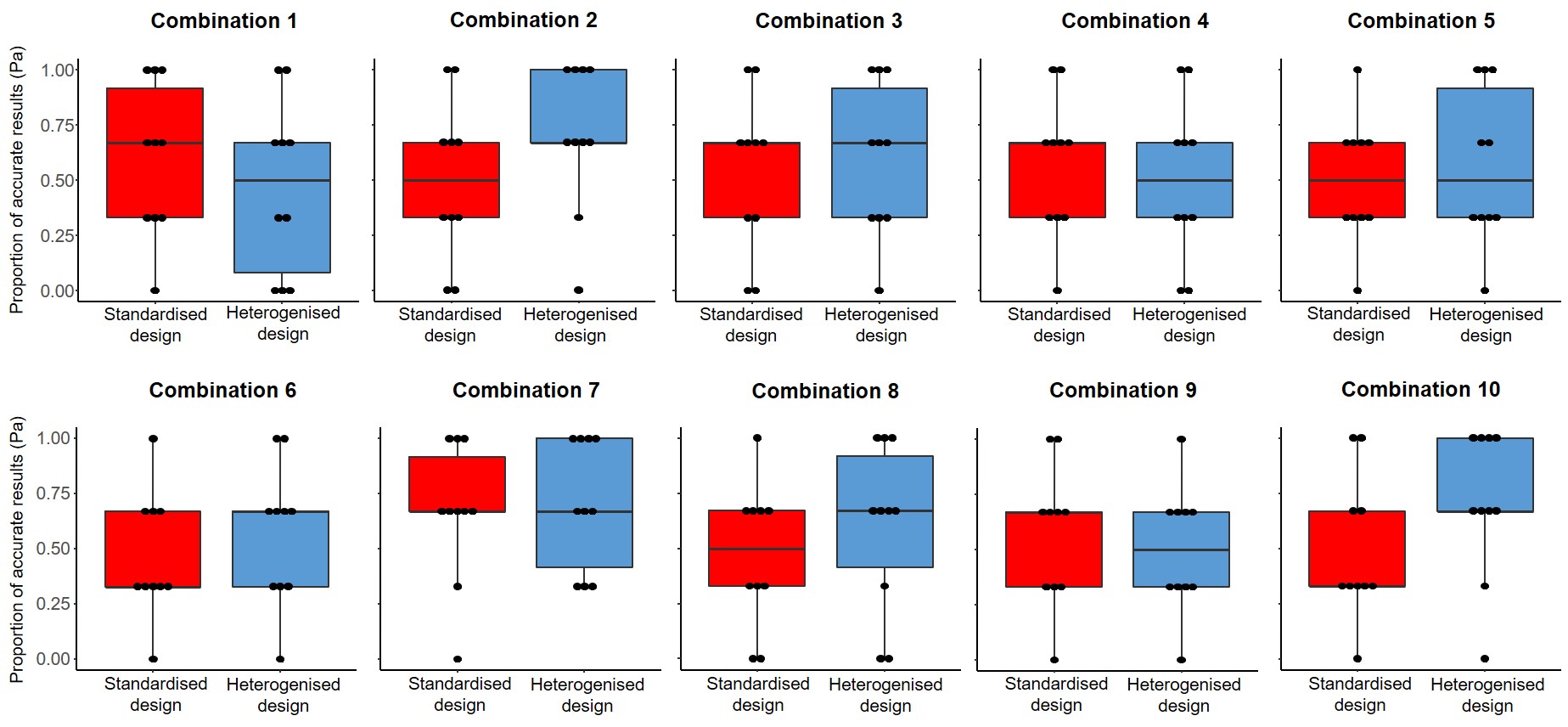

Supplement: S3 Fig — Shown are Pa ratios of 10 outcome measures for 10 alternative allocations of the experimenters to the designs (Combination 1–10). Data are presented as boxplots showing medians, 25% and 75% percentiles, and 5% and 95% percentiles. Black dots represent single values for each outcome measure in both designs. Statistics: Wilcoxon signed-rank test (paired, one-tailed, n = 10). The raw and processed data underlying this figure are available in the Figshare repositories https://figshare.com/s/f327175aa8b541ef01bd and https://figshare.com/s/2245cee43a544ee1ffff. Pa, proportion of accurate results. (JPG) [file pbio.3001564.s014.jpg]

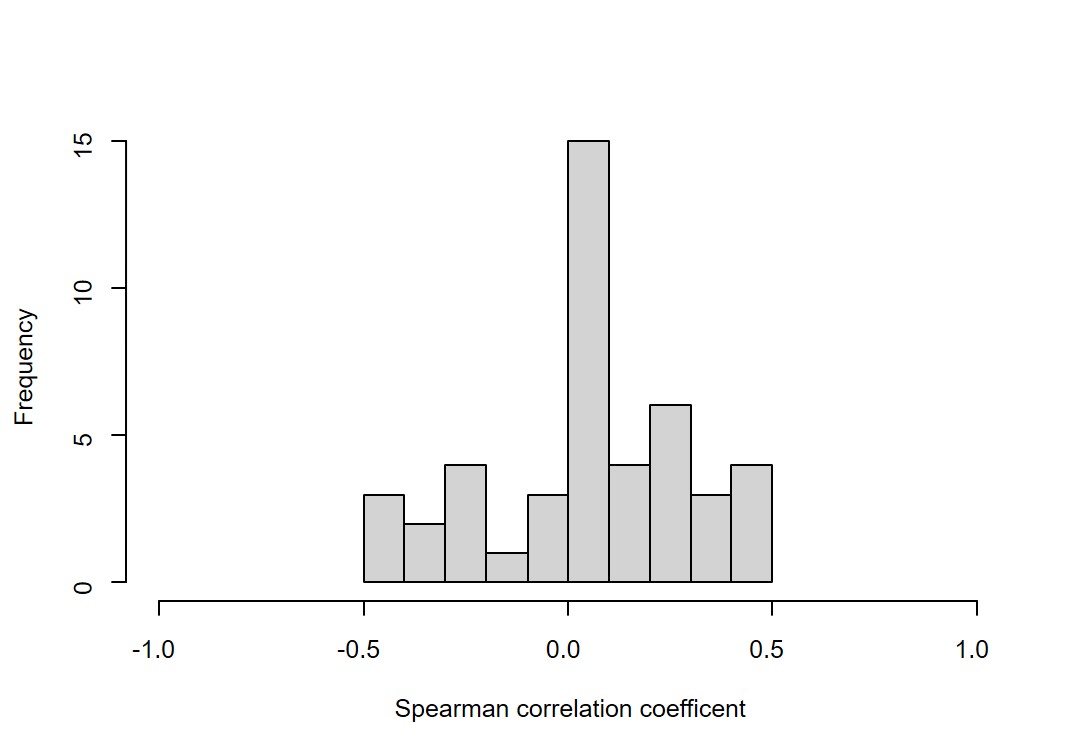

Supplement: S4 Fig — The raw data underlying this figure are available in the Figshare repository https://figshare.com/s/f327175aa8b541ef01bd. (JPG) [file pbio.3001564.s015.jpg]

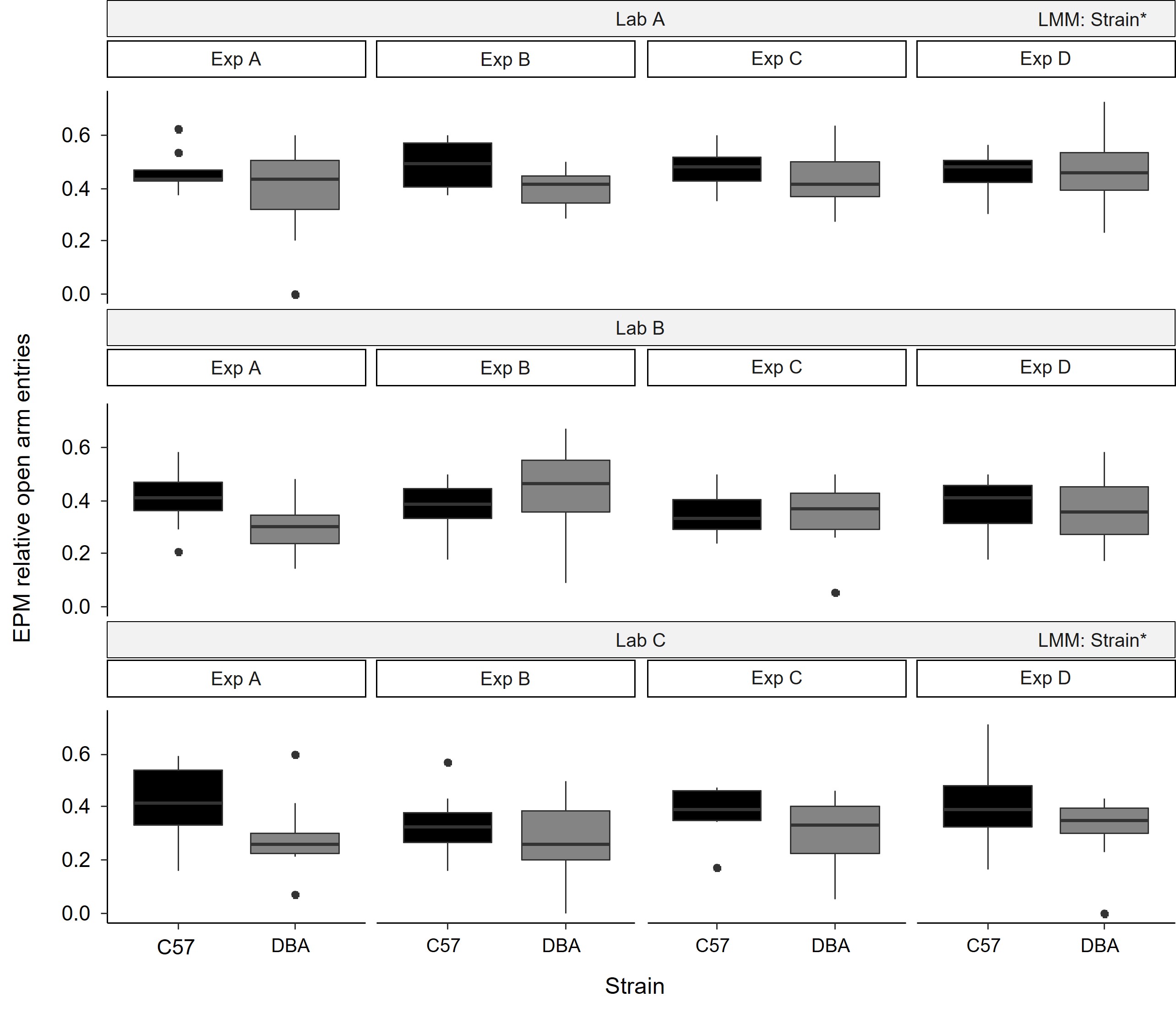

Supplement: S5 Fig — Results are displayed separately for each experimenter (Exp A–Exp D) conducting a full experiment (n = 12) in each laboratory. Data are presented as boxplots showing medians, 25% and 75% percentiles, and 5% and 95% percentiles. Statistics: LMMs followed by Tukey’s test for post hoc pairwise comparisons of the means. The analyses were conducted separately for the data of each laboratory, * p < 0.05. Abbreviations are indicating a significant strain effect (Strain*), experimenter effect (Exp*), or strain-by-experimenter interaction (Strain × Exp*). The raw data underlying this figure are available in the Figshare repository https://figshare.com/s/f327175aa8b541ef01bd. EPM, Elevated Plus Maze; LMM, linear mixed model. (JPG) [file pbio.3001564.s016.jpg]

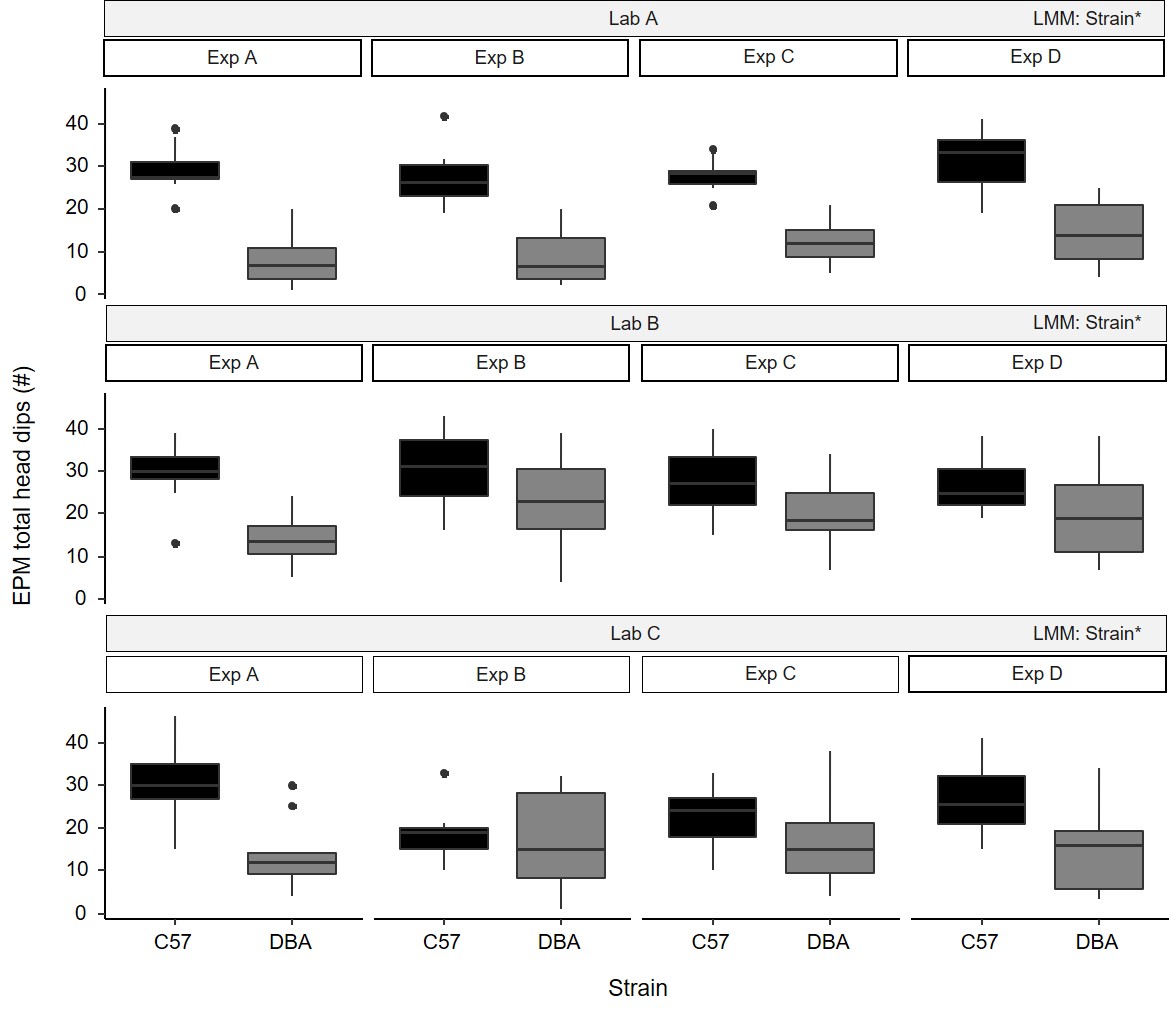

Supplement: S6 Fig — Results are displayed separately for each experimenter (Exp A–Exp D) conducting a full experiment (n = 12) in each laboratory. Data are presented as boxplots showing medians, 25% and 75% percentiles, and 5% and 95% percentiles. Statistics: LMMs followed by Tukey’s test for post hoc pairwise comparisons of the means. The analyses were conducted separately for the data of each laboratory, * p < 0.05. Abbreviations are indicating a significant strain effect (Strain*), experimenter effect (Exp*), or strain-by-experimenter interaction (Strain × Exp*). The raw data underlying this figure are available in the Figshare repository https://figshare.com/s/f327175aa8b541ef01bd. EPM, Elevated Plus Maze; LMM, linear mixed model. (JPG) [file pbio.3001564.s017.jpg]

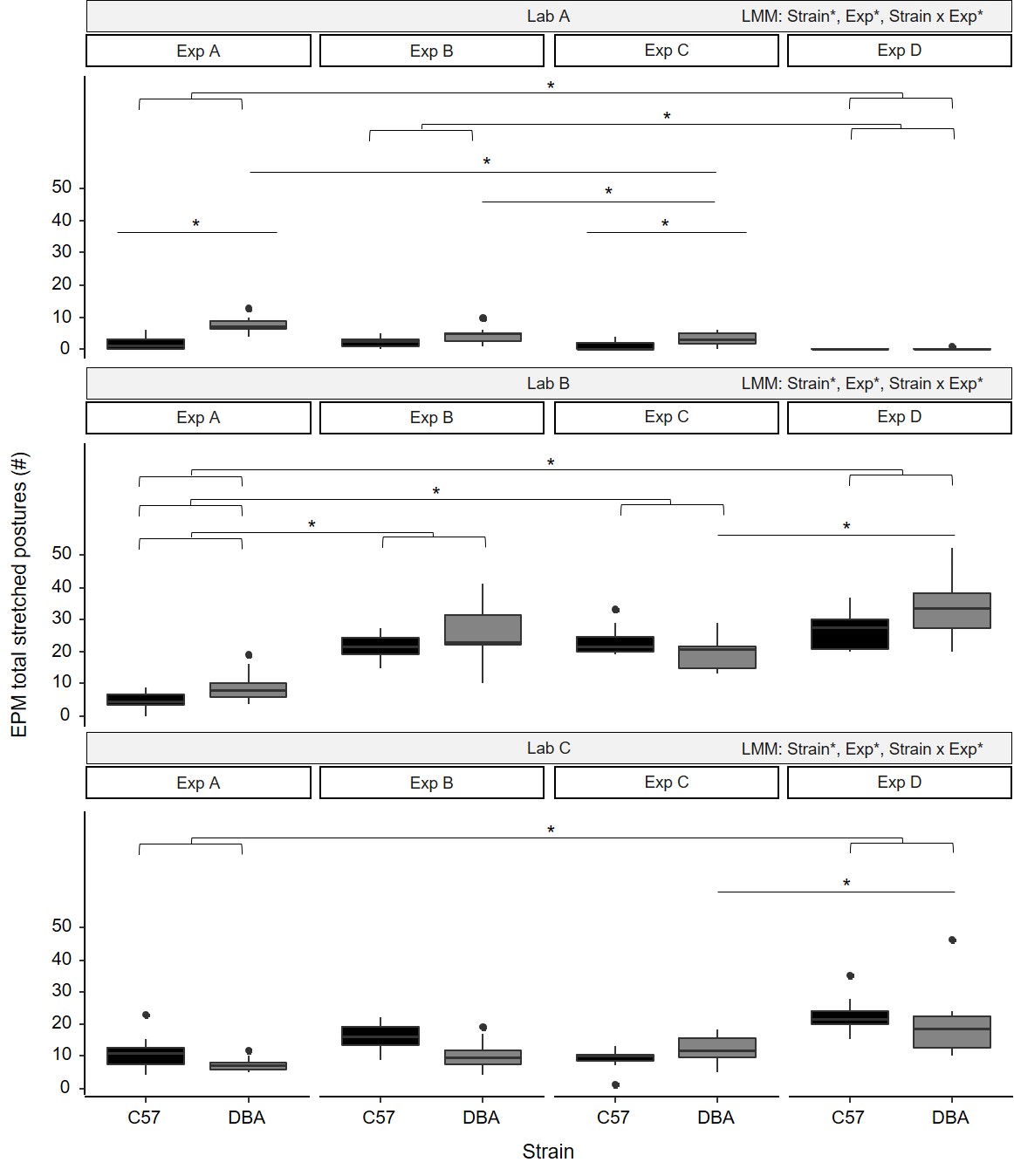

Supplement: S7 Fig — Results are displayed separately for each experimenter (Exp A–Exp D) conducting a full experiment (n = 12) in each laboratory. Data are presented as boxplots showing medians, 25% and 75% percentiles, and 5% and 95% percentiles. Statistics: LMMs followed by Tukey’s test for post hoc pairwise comparisons of the means. The analyses were conducted separately for the data of each laboratory, * p < 0.05. Abbreviations are indicating a significant strain effect (Strain*), experimenter effect (Exp*), or strain-by-experimenter interaction (Strain × Exp*). The raw data underlying this figure are available in the Figshare repository https://figshare.com/s/f327175aa8b541ef01bd. EPM, Elevated Plus Maze; LMM, linear mixed model. (JPG) [file pbio.3001564.s018.jpg]

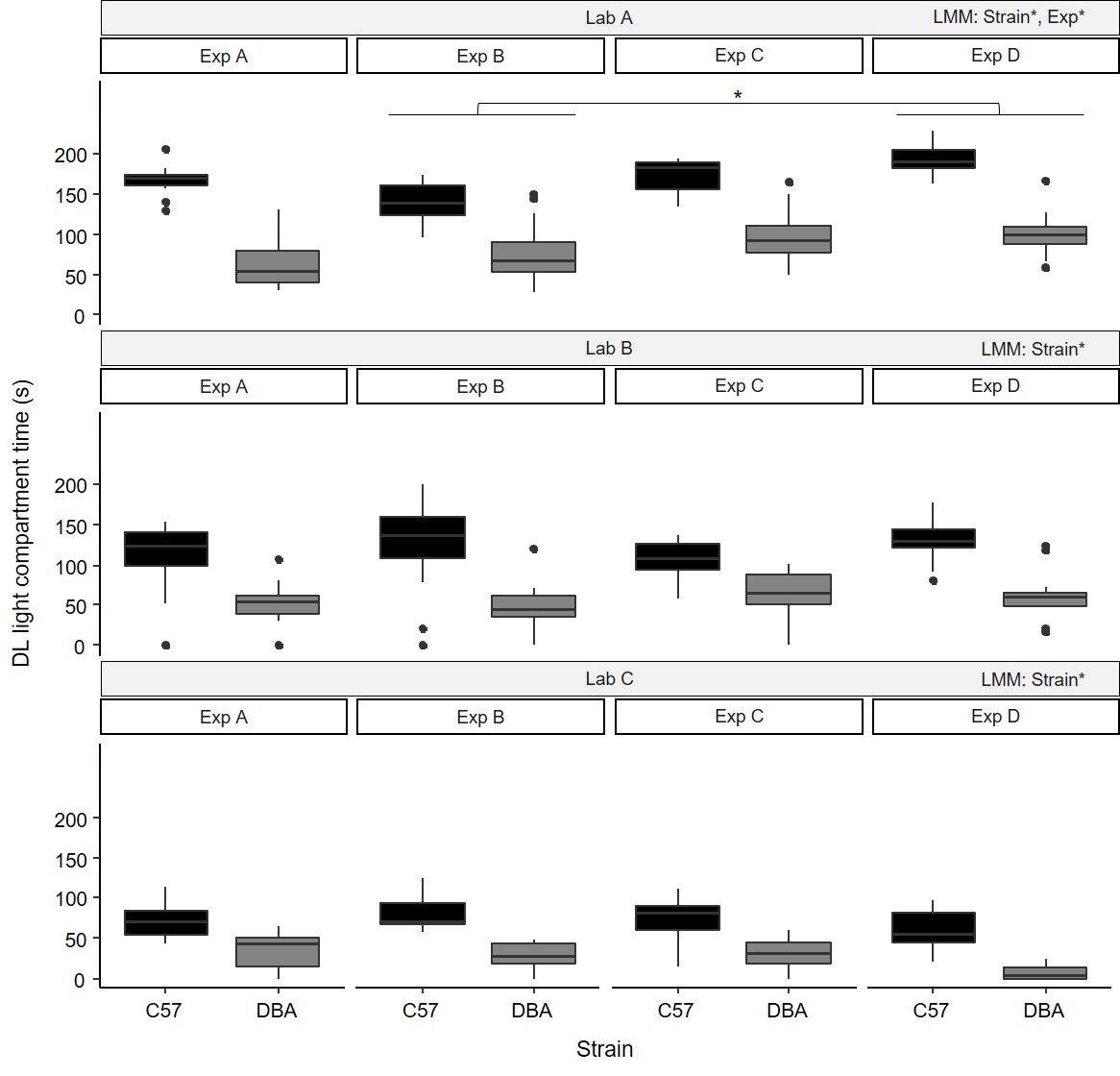

Supplement: S8 Fig — Results are displayed separately for each experimenter (Exp A–Exp D) conducting a full experiment (n = 12) in each laboratory. Data are presented as boxplots showing medians, 25% and 75% percentiles, and 5% and 95% percentiles. Statistics: LMMs followed by Tukey’s test for post hoc pairwise comparisons of the means. The analyses were conducted separately for the data of each laboratory, * p < 0.05. Abbreviations are indicating a significant strain effect (Strain*), experimenter effect (Exp*), or strain-by-experimenter interaction (Strain × Exp*). The raw data underlying this figure are available in the Figshare repository https://figshare.com/s/f327175aa8b541ef01bd. DL, Dark Light; LMM, linear mixed model. (JPG) [file pbio.3001564.s019.jpg]

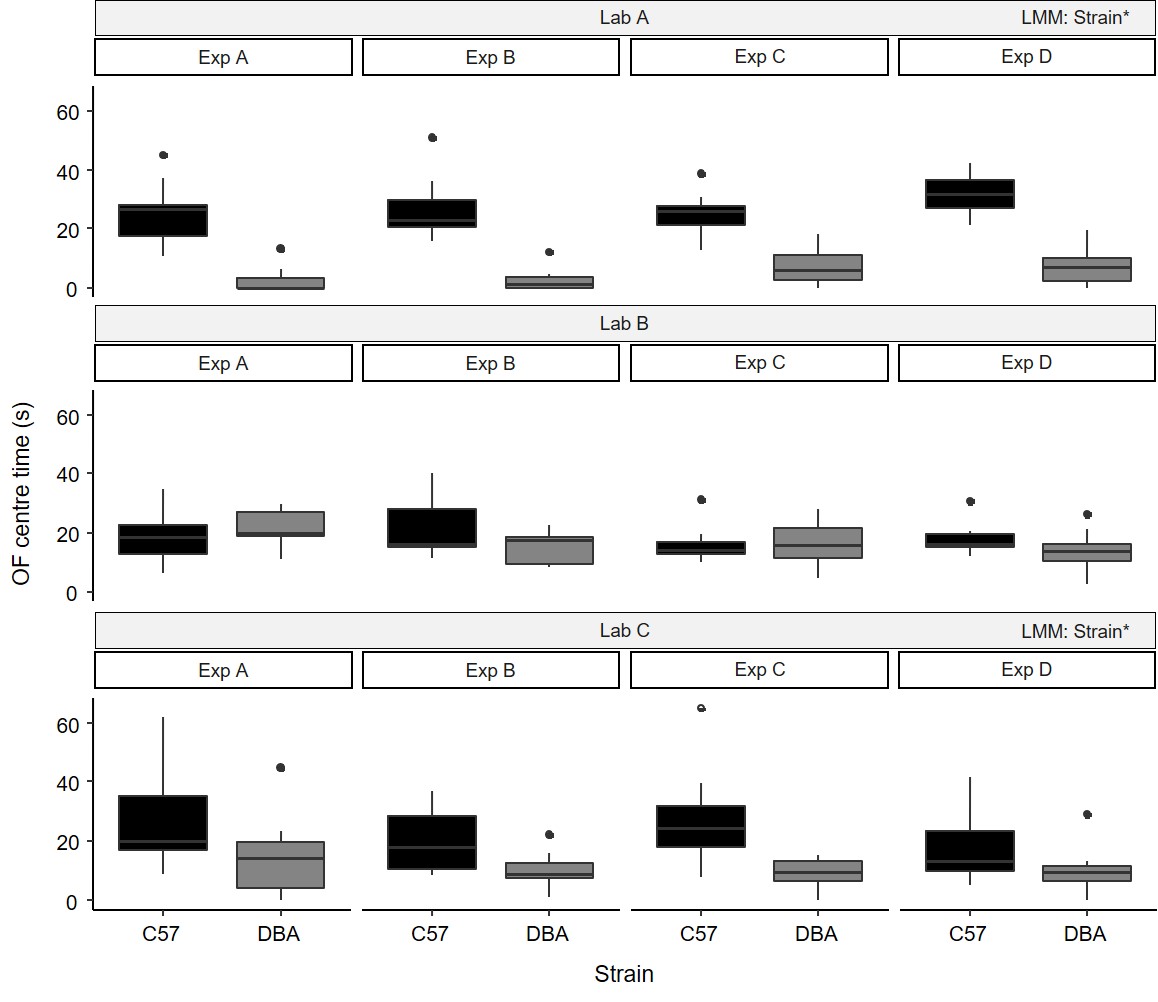

Supplement: S9 Fig — Results are displayed separately for each experimenter (Exp A–Exp D) conducting a full experiment (n = 12) in each laboratory. Data are presented as boxplots showing medians, 25% and 75% percentiles, and 5% and 95% percentiles. Statistics: LMMs followed by Tukey’s test for post hoc pairwise comparisons of the means. The analyses were conducted separately for the data of each laboratory, * p < 0.05. Abbreviations are indicating a significant strain effect (Strain*), experimenter effect (Exp*), or strain-by-experimenter interaction (Strain × Exp*). The raw data underlying this figure are available in the Figshare repository https://figshare.com/s/f327175aa8b541ef01bd. LMM, linear mixed model; OF, Open Field. (JPG) [file pbio.3001564.s020.jpg]

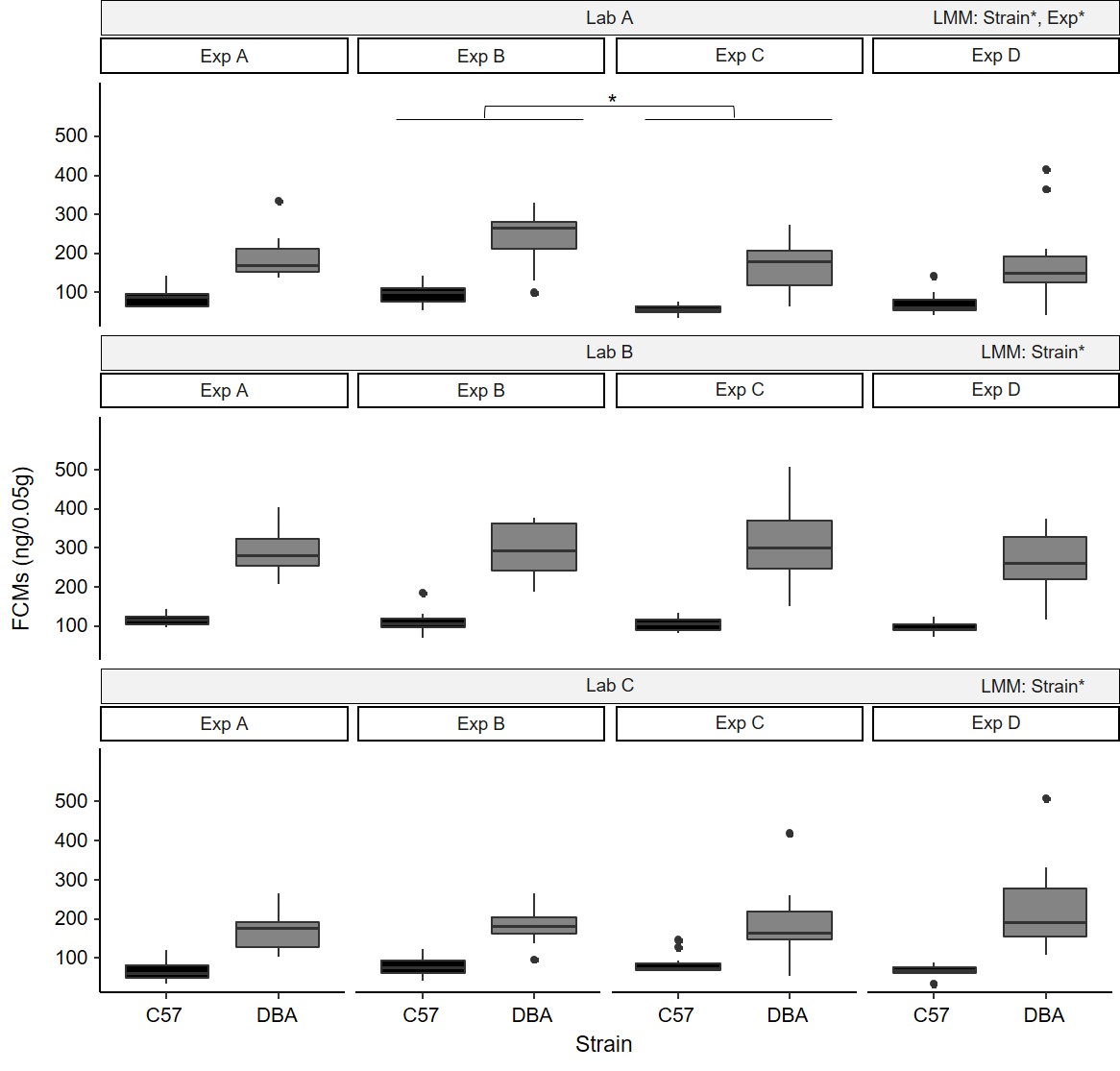

Supplement: S10 Fig — Results are displayed separately for each experimenter (Exp A–Exp D) conducting a full experiment (n = 12) in each laboratory. Data are presented as boxplots showing medians, 25% and 75% percentiles, and 5% and 95% percentiles. Statistics: LMMs followed by Tukey’s test for post hoc pairwise comparisons of the means. The analyses were conducted separately for the data of each laboratory, * p < 0.05. Abbreviations are indicating a significant strain effect (Strain*), experimenter effect (Exp*), or strain-by-experimenter interaction (Strain × Exp*). The raw data underlying this figure are available in the Figshare repository https://figshare.com/s/f327175aa8b541ef01bd. FCMs, faecal corticosterone metabolites; LMM, linear mixed model. (JPG) [file pbio.3001564.s021.jpg]

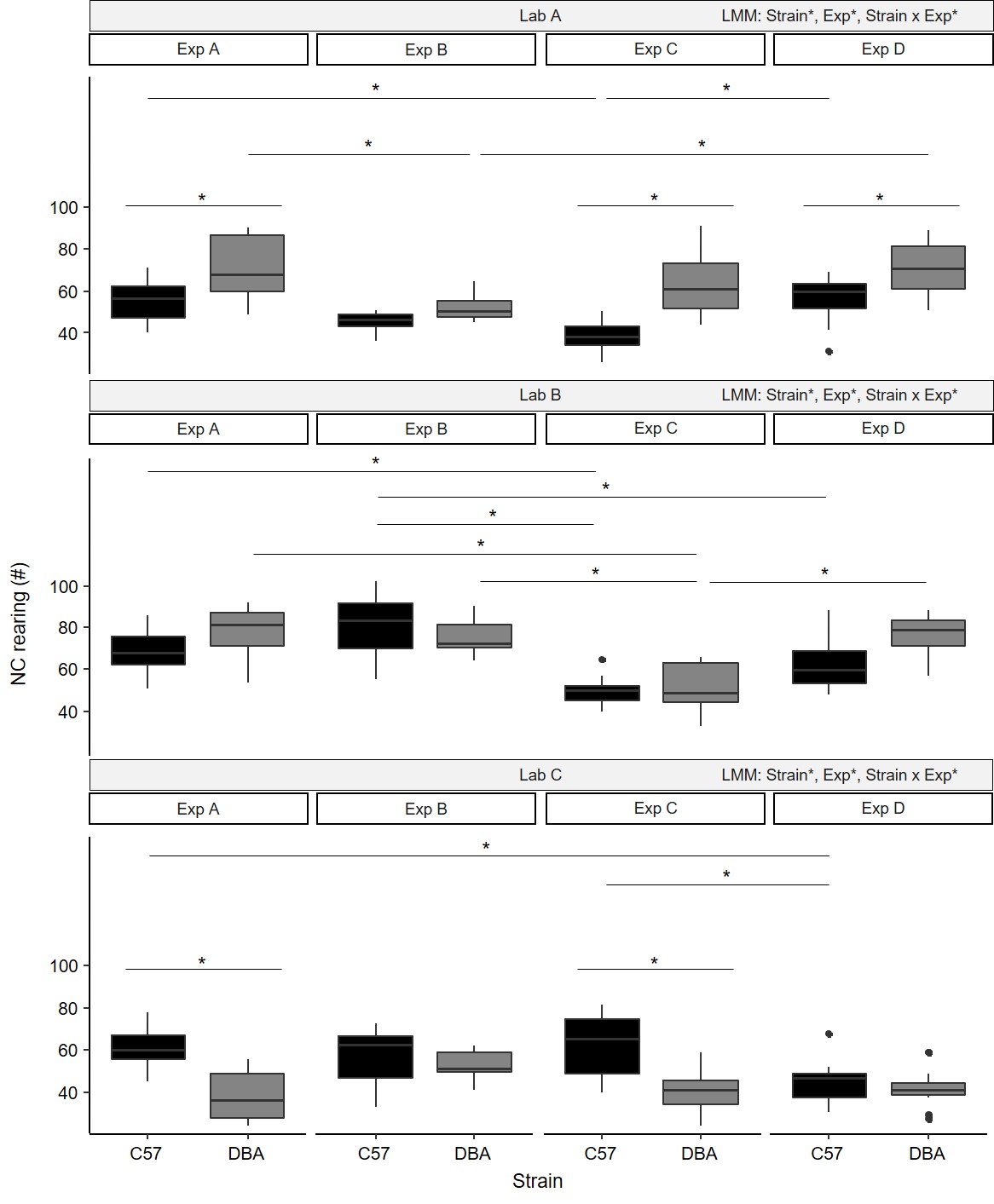

Supplement: S11 Fig — Results are displayed separately for each experimenter (Exp A–Exp D) conducting a full experiment (n = 12) in each laboratory. Data are presented as boxplots showing medians, 25% and 75% percentiles, and 5% and 95% percentiles. Statistics: LMMs followed by Tukey’s test for post hoc pairwise comparisons of the means. The analyses were conducted separately for the data of each laboratory, * p < 0.05. Abbreviations are indicating a significant strain effect (Strain*), experimenter effect (Exp*), or strain-by-experimenter interaction (Strain × Exp*). The raw data underlying this figure are available in the Figshare repository https://figshare.com/s/f327175aa8b541ef01bd. LMM, linear mixed model; NC, Novel Cage. (JPG) [file pbio.3001564.s022.jpg]

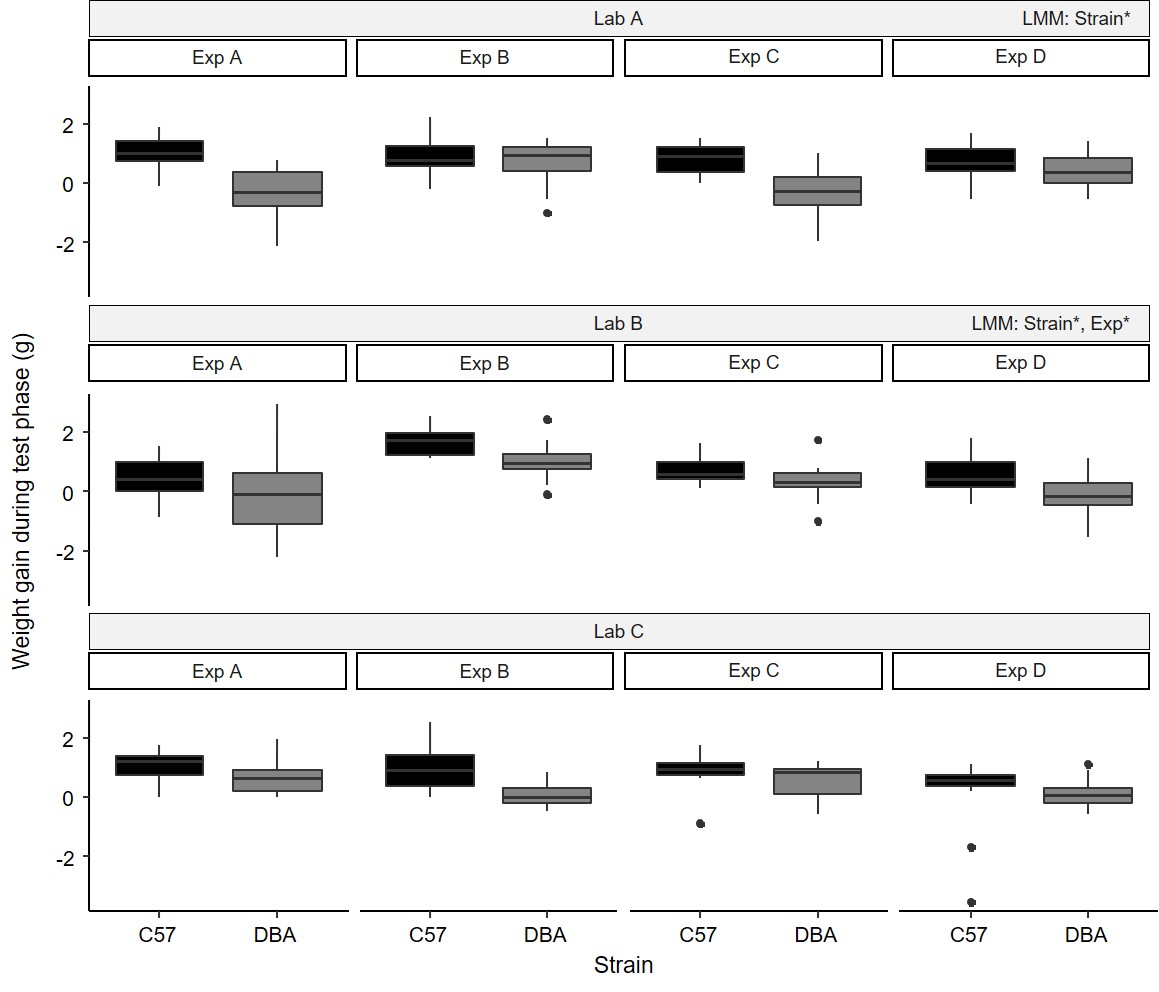

Supplement: S12 Fig — Results are displayed separately for each experimenter (Exp A–Exp D) conducting a full experiment (n = 12) in each laboratory. Data are presented as boxplots showing medians, 25% and 75% percentiles, and 5% and 95% percentiles. Statistics: LMMs followed by Tukey’s test for post hoc pairwise comparisons of the means. The analyses were conducted separately for the data of each laboratory, * p < 0.05. Abbreviations are indicating a significant strain effect (Strain*), experimenter effect (Exp*), or strain-by-experimenter interaction (Strain × Exp*). The raw data underlying this figure are available in the Figshare repository https://figshare.com/s/f327175aa8b541ef01bd. LMM, linear mixed model. (JPG) [file pbio.3001564.s023.jpg]

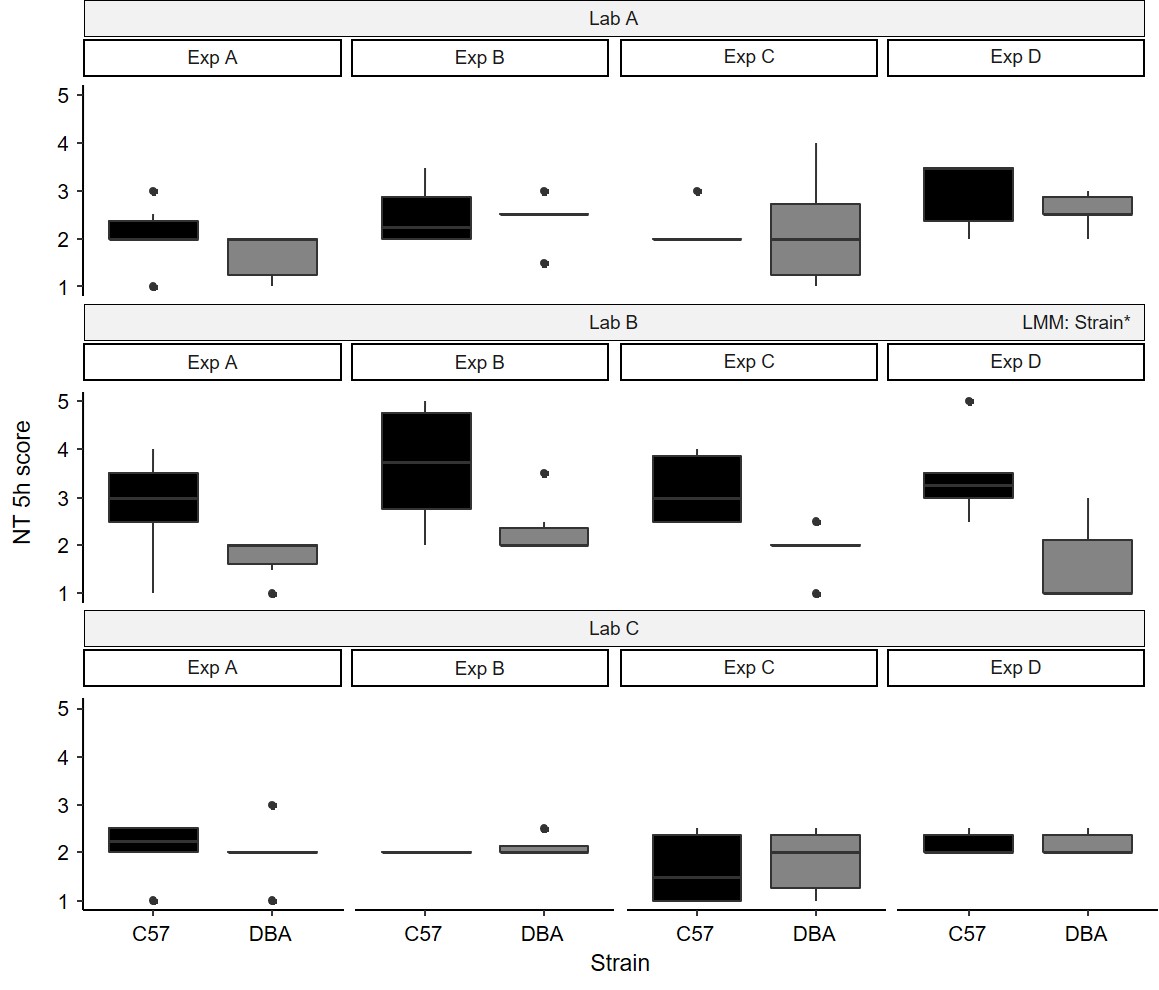

Supplement: S13 Fig — Results are displayed separately for each experimenter (Exp A–Exp D) conducting a full experiment (n = 12) in each laboratory. Data are presented as boxplots showing medians, 25% and 75% percentiles, and 5% and 95% percentiles. Statistics: LMMs followed by Tukey’s test for post hoc pairwise comparisons of the means. The analyses were conducted separately for the data of each laboratory, * p < 0.05. Abbreviations are indicating a significant strain effect (Strain*), experimenter effect (Exp*), or strain-by-experimenter interaction (Strain × Exp*). The raw data underlying this figure are available in the Figshare repository https://figshare.com/s/f327175aa8b541ef01bd. LMM, linear mixed model; NT, Nest. (JPG) [file pbio.3001564.s024.jpg]

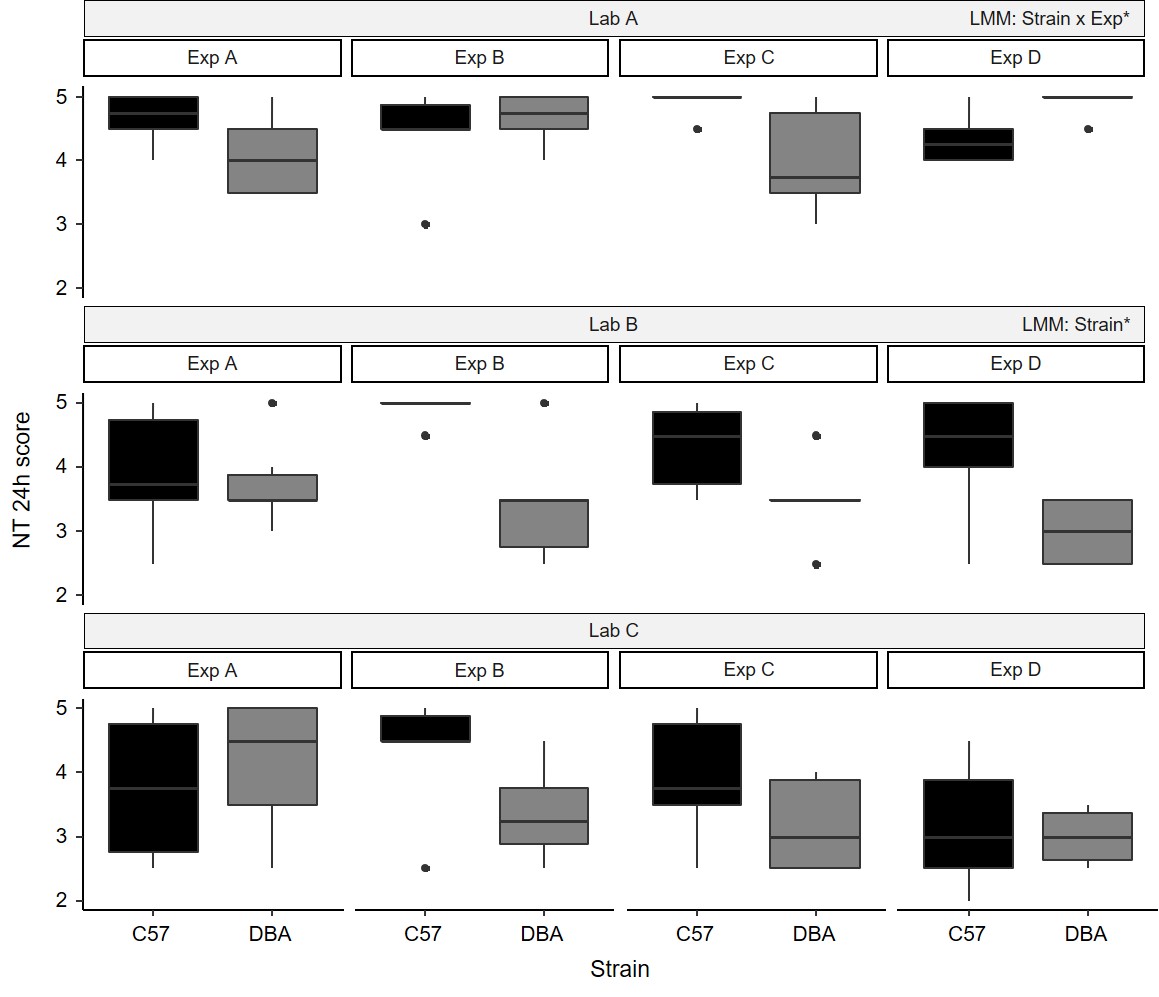

Supplement: S14 Fig — Results are displayed separately for each experimenter (Exp A–Exp D) conducting a full experiment (n = 12) in each laboratory. Data are presented as boxplots showing medians, 25% and 75% percentiles, and 5% and 95% percentiles. Statistics: LMMs followed by Tukey’s test for post hoc pairwise comparisons of the means. The analyses were conducted separately for the data of each laboratory, * p < 0.05. Abbreviations are indicating a significant strain effect (Strain*), experimenter effect (Exp*), or strain-by-experimenter interaction (Strain × Exp*). The raw data underlying this figure are available in the Figshare repository https://figshare.com/s/f327175aa8b541ef01bd. LMM, linear mixed model; NT, Nest. (JPG) [file pbio.3001564.s025.jpg]
